# Genetic diversity promotes resilience in a mouse model of Alzheimer's disease

**DOI:** 10.1002/alz.13753

**Published:** 2024-03-01

**Authors:** Neelakshi Soni, Lindsay A. Hohsfield, Kristine M. Tran, Shimako Kawauchi, Amber Walker, Dominic Javonillo, Jimmy Phan, Dina Matheos, Celia Da Cunha, Asli Uyar, Giedre Milinkeviciute, Angela Gomez‐Arboledas, Katelynn Tran, Catherine C. Kaczorowski, Marcelo A. Wood, Andrea J. Tenner, Frank M. LaFerla, Gregory W. Carter, Ali Mortazavi, Vivek Swarup, Grant R. MacGregor, Kim N. Green

**Affiliations:** ^1^ Department of Neurobiology and Behavior University of California Irvine California USA; ^2^ Institute for Memory Impairments and Neurological Disorders University of California Irvine California USA; ^3^ Transgenic Mouse Facility, ULAR Office of Research University of California Irvine California USA; ^4^ The Jackson Laboratory Bar Harbor Maine USA; ^5^ Department of Neurology University of Michigan Ann Arbor Michigan USA; ^6^ Department of Molecular Biology and Biochemistry University of California Irvine California USA; ^7^ Department of Pathology and Laboratory Medicine University of California Irvine California USA; ^8^ Department of Developmental and Cellular Biology University of California Irvine California USA; ^9^ Center for Complex Biological Systems University of California Irvine California USA

**Keywords:** 5xFAD, Alzheimer's disease, amyloid, astrocytes, collaborative cross mice, genetic diversity, microglia, neurofilament light chain, resilience

## Abstract

**INTRODUCTION:**

Alzheimer's disease (AD) is a neurodegenerative disorder with multifactorial etiology, including genetic factors that play a significant role in disease risk and resilience. However, the role of genetic diversity in preclinical AD studies has received limited attention.

**METHODS:**

We crossed five Collaborative Cross strains with 5xFAD C57BL/6J female mice to generate F1 mice with and without the 5xFAD transgene. Amyloid plaque pathology, microglial and astrocytic responses, neurofilament light chain levels, and gene expression were assessed at various ages.

**RESULTS:**

Genetic diversity significantly impacts AD‐related pathology. Hybrid strains showed resistance to amyloid plaque formation and neuronal damage. Transcriptome diversity was maintained across ages and sexes, with observable strain‐specific variations in AD‐related phenotypes. Comparative gene expression analysis indicated correlations between mouse strains and human AD.

**DISCUSSION:**

Increasing genetic diversity promotes resilience to AD‐related pathogenesis, relative to an inbred C57BL/6J background, reinforcing the importance of genetic diversity in uncovering resilience in the development of AD.

**Highlights:**

- Genetic diversity's impact on AD in mice was explored.
- Diverse F1 mouse strains were used for AD study, via the Collaborative Cross.
- Strain‐specific variations in AD pathology, glia, and transcription were found.
- Strains resilient to plaque formation and plasma neurofilament light chain (NfL) increases were identified.
- Correlations with human AD transcriptomics were observed.

## 1 BACKGROUND

Alzheimer's disease (AD) is a progressive neurodegenerative disorder characterized by the accumulation of amyloid beta (Aβ) plaques and tau neurofibrillary tangles.^1^ While age is the primary risk factor for AD, genetics has a substantial impact on disease susceptibility and resilience.^2^ Twin studies estimate that AD has a heritability of 50% to 80%, although much of the genetic risk and variability associated with AD remains unexplained.^3^, ^4^

Familial AD (FAD) is a rare autosomal dominant disorder caused by pathological variants in *APP*, *PSEN1*, and *PSEN2* genes,^5^ while late‐onset AD (LOAD) exhibits a much more complex genetic architecture.^6^, ^7^ Genome‐wide association studies (GWAS) have expanded our knowledge about potential sources of this complexity, with >40 genetic risk variants having been identified for LOAD, including *APOE*, *BIN1*, *CLU*, *CR1*, *PICALM*, and *TREM2*.^8^, ^9^, ^10^, ^11^ Except for *APOE* and *TREM2*, risk variants have a relatively small effect size on AD risk,^10^, ^12^, ^13^ but the accumulation of multiple variants may increase susceptibility to AD risk. These GWAS LOAD‐risk variants appear to converge on pathways associated with amyloid and tau/protein catabolism, cholesterol/lipid metabolism, endocytosis/phagocytosis, and innate immunity^14^, ^15^ and support the idea that LOAD is likely polygenic.^16^, ^17^, ^18^

Due to similarities in their genetics and physiology, mice serve as important models for understanding the biological processes underlying human disease and have been used for many decades in AD research.^19^ Historically, most transgenic AD mouse models have introduced FAD mutations into the genetic background of a single inbred strain, for example, C57BL/6J (B6J), or into hybrid backgrounds, for example, two strains such as B6J and SJL.^20^ Such mouse strains have limited genetic variation and fail to capture the genetic diversity found in the human population. In recognition of the importance of genetic diversity as a key factor when optimizing AD mouse models and to better recapitulate the human disease, researchers are now developing models that incorporate genetic variation.

Previous studies have shown that genetic variation can modify the impact of FAD mutations on AD phenotypes.^2^, ^19^, ^21^, ^22^, ^23^, ^24^, ^25^ AD transgenic mice on different inbred strain backgrounds exhibit modified parenchymal Aβ.^21^, ^22^, ^23^, ^24^ Neuner et al. generated an AD‐BXD transgenic mouse panel and showed that genetic background significantly affected cognitive function, Aβ levels, and risk gene expression.^2^ Onos et al. incorporated FAD mutations into genetically distinct, wild‐derived mouse strains and showed that inclusion of genetic variation significantly altered the immune response to amyloid and AD pathology.^26^

In addition to better recapitulating the disease, new mouse genetics resources enable mapping of complex traits with higher resolution. One such resource are the genetically diverse recombinant inbred mouse Collaborative Cross (CC) strains. CC strains were generated by crossing eight genetically diverse inbred founder strains that together capture around 90% of the genetic diversity observed in *Mus musculus* species. Five common laboratory (A/J, C57BL/6J, 129S1/SvImJ, NOD/ShiLtJ, NZO/HlLtJ) and three wild‐derived inbred (CAST/EiJ, PWK/PhJ, and WSB/EiJ) strains were crossed using a funnel breeding scheme that included all eight parental strains, followed by inbreeding, to produce a panel of recombinant inbred strains with different contributions from the eight founder strains. The genomes of 69 CC lines have been sequenced and reconstructed from the founders with an average of ∼6 M single nucleotide polymorphisms (SNPs) per strain and a combined 43 million SNPs, of which 40 million correspond to the reported SNPs of the founder strains.^27^ The genetic diversity in the extant CC strains better approximates the complex genetic makeup of the human population.^28^, ^29^, ^30^

To model the role of genetic diversity in human AD, we generated a series of F1 hybrid mice by crossing the 5xFAD transgenic line, a well‐known model of amyloidosis,^31^ to five different CC strains, resulting in different allelic contributions: non‐transgenic (wild type [WT]) B6CCF1 (C57BL/6J x CC)F1 and 5x‐B6CCF1 (5xFAD hemizygous C57BL/6J x CC)F1. Here, we present a detailed analysis of transcriptional, immunohistochemical, and biochemical data from this series of mice to investigate the effect of increased genetic diversity on AD pathophysiology in a mouse model of AD.

## 2 METHODS

### 2.1 Mice

Collaborative Cross (CC) male mice used in this study were obtained from The Jackson Laboratory (Bar Harbor, ME, USA). Before transfer to JAX, CC lines were generated and bred at Tel Aviv University in Israel, Geniad in Australia, and Oak Ridge National Laboratory in the USA. A funnel breeding scheme was used to generate recombinant inbred strains with different contributions from the eight founder strains (A/J, C57BL/6J, 129S1/SvImJ, NOD/ShiLtJ, NZO/HlLtJ, CAST/EiJ, PWK/PhJ, and WSB/EiJ). Inbreeding depression, infertility, and additional issues led to the attrition of most strains. Despite these limitations, around 70 CC strains are currently available for experimental use. The mice used in this study were 4‐ to 12‐week‐old male C57BL/6J (B6J), CC002/Unc (CC002), CC006/TauUnc (CC006), CC013/GeniUnc (CC013), CC017/Unc (CC017), and CC037/TauUnc (CC037), plus female 5xFAD hemizygous mice (B6.CgTg(APPSwFlLon,PSEN1*M146L*L286V) 6799Vas/Mmjax, Stock no. 034848‐JAX, MMRRC). The five CC lines were selected based on the diversity of expression (ie, transcription) of AD risk‐related genes (*APOE, BIN1, CLU, ABCA7, PICALM, CD33, CD2AP, TREM2, IL1RAP, MECP2, SORL1, TYROBP*)^32^ as these would be expected to affect AD‐related pathology. The CC lines used were also selected based on breeding ability and availability. All animals were bred by the Transgenic Mouse Facility at the University of California, Irvine (UCI). Male CC mice were bred with female hemizygous 5xFAD animals on a congenic B6J background to produce F1 progeny of which 50% contained the 5xFAD transgene (hereafter referred to as 5x‐CCnnn, where nnn is the CC strain number), while the other 50% of littermates were used as non‐transgenic controls (hereafter referred to as WT‐CCnnn). After weaning, mice were housed together with littermates and aged until harvest. Mice were housed in Super Mouse 750 ventilated cages (Lab Products, Inc., Seaford, DE, USA) with 70 air changes per hour. Cages contained 3.175‐mm (0.125‐in.) corn cob bedding and two cotton nestlet squares (6 g) for bedding. Light cycle was 14 h on/10 h off (lights on at 6:30 a.m., off at 8:30 p.m.), room temperature was set at 72°F with a variance of ± 2 °F. A standard food diet (LabDiet mouse Irr 6F [7.4% fat] and autoclaved acidified water (pH 2.5 to 3.0) were provided ad libitum.

Assignment of animals to treatment groups was conducted in a random manner and was balanced for sex. Researchers were blinded to genotype and treatment groups during analysis.

**RESEARCH IN CONTEXT**

1. **Systematic review**: AD pathology is influenced by genetic factors, yet the impact of genetic diversity on disease progression remains underexplored. Previous studies primarily focused on singular genetic models, limiting the understanding of genetic variations in AD. Our research addresses this gap by investigating the influence of genetic diversity on AD pathology using a panel of genetically diverse F1 mouse strains.
2. **Interpretation**: Our study utilized a novel approach by crossing different Collaborative Cross (CC) lines with C57BL/6J mice, both with and without the 5xFAD transgene, to investigate the role of genetic diversity in AD progression. We observed that genetic diversity significantly affects amyloid plaque pathology, neuronal damage, microglial and astrocytic responses, and neurofilament light chain (NfL) levels. The study revealed strain‐specific variations in AD‐related phenotypes and highlighted correlations between mouse models and human AD pathology.
3. **Future directions**: Future research should focus on disentangling the genetic contributions to AD resiliency and susceptibility. This includes a deeper exploration of gene expression variations among different strains and their correlation with human AD. Additionally, developing targeted therapeutic strategies based on the genetic backgrounds that confer resistance to AD pathology could be highly beneficial. Understanding the molecular mechanisms underpinning these genetic influences will further enhance the development of precision medicine approaches in AD treatment and prevention.

### 2.2 Animal treatment

All animal experiments were performed according to animal protocols approved by the Institutional Animal Care and Use Committee at UCI, an American Association for Accreditation of Laboratory Animal Care (AAALAC)‐accredited institution. Four‐ and 12‐month‐old mice were euthanized using CO_2_ inhalation, then transcardially perfused with 1x phosphate‐buffered saline (PBS). The brains were collected and divided into hemispheres; one was flash frozen for biochemical analysis or RNA sequencing (RNA‐seq), and the other was drop‐fixed in 4% paraformaldehyde (PFA; Thermo Fisher Scientific, Waltham, MA, USA) for immunohistochemical analysis. Fixed brains were cryopreserved in PBS + 0.05% sodium azide + 30% sucrose, and a Leica SM2000R freezing microtome was used to generate 40‐μm‐thick coronal brain slices. The coronal brain slices were collected between −2.78 mm posterior and −3.38 mm posterior to Bregma according to the Allen Mouse Brain Atlas (Reference atlas version 1, 2008). Brain sections were stored in 30% glycerol + 30% ethyl glycol in 1x PBS at −20°C until histological evaluation.

### 2.3 Immunohistochemistry

Fluorescent immunostaining was done using a standard indirect technique as previously described.^33^, ^34^, ^35^ Mouse brain free‐floating sections belonging to the same experimental group (same genotype, age, and sex) were pooled into a single well during the immunostaining process. For amyloid staining, brain sections were washed once for 5 min in 1x PBS and then dehydrated in a series of graded alcohol dilutions (100%, 95%, 70%, 50%; 1 × 3 min each). Following this, the sections were incubated in 0.5% Thioflavin‐S (ThioS;1892; Sigma‐Aldrich, St. Louis, MO, USA) and diluted in 50% ethanol for 10 min. The sections were then washed three times with 50% ethanol for 5 min and then once with 1x PBS for 10 min. To avoid loss of signal, the sections were kept in the dark during the incubation periods after ThioS. For subsequent immunohistochemical staining, brain sections were immersed for 1 h in normal blocking serum (5% normal goat serum with 0.2% Triton X‐100 in 1x PBS). The primary antibodies and dilutions used to stain brain sections are as follows: Aβ1‐16 (6E10; 1:2000; 8030001; BioLegend, San Diego, CA, USA), ionized calcium‐binding adapter molecule 1 (IBA1; 1:2000; 019‐19741; Wako, Osaka, Japan), glial fibrillary acidic protein (GFAP; 1:1000; AB134436; Abcam, Cambridge, MA, USA), S100β (1:200; AB41548; Abcam, Cambridge, MA, USA). Brain sections were incubated overnight in the primary antibody at the dilutions described above in normal serum blocking solution at 4°C. The next day, sections were washed three times in 1x PBS for 5 min before incubation in a fluorescent dye (Alexa Fluor)‐conjugated secondary antibody in normal serum blocking solution (1:200 for all species and wavelengths; Invitrogen) for 1 h. Once the staining was complete, sections were washed three times in 1x PBS for 5 min before being mounted and coverslipped. Whole‐brain stitches of the brain sections were obtained using an automatic slide scanner (Zeiss AxioScan.Z1) using a 10 × 0.45NA Plan‐Apo objective, equipped with an AxioCam MRm camera and Zen AxioScan 2.3 software. High‐resolution fluorescence confocal images of brain sections were captured at 20× using a Leica TCS SPE‐II confocal microscope and LAS‐X software. For image analysis, a single field of view (FOV) per brain region (somatosensory cortex and subiculum) per mouse was captured using the Allen Brain Atlas. Comparable sections of tissue from each animal were imaged using a 20× objective, at multiple *z*‐planes, except for IBA1 images from 12‐month‐old animals, which were a single plane.^33^, ^35^

### 2.4 Imaris quantitative analysis

Brain sections were stained at the same time so that all quantitative comparisons could be made between experimental groups. Image quantification analysis was done using Bitplane Imaris version 9.7 software (Biplane Inc., Zürich, Switzerland). Automatic quantification of cells (ie, microglia and astrocytes) was performed using the spots module within Imaris, then normalized to the area of the FOV. ThioS^+^ plaque numbers were quantified using the spots module then normalized to the FOV area. All volumetric measurements (eg, ThioS^+^ plaque volume, IBA1^+^ microglia volume) were quantified utilizing the surfaces module. Plaque load was calculated by normalizing the total volume of ThioS^+^ plaques to the FOV volume.

### 2.5 Soluble and insoluble fraction Aβ quantification

Sample preparation and quantification of Aβ was performed as described.^33^, ^35^ Brain hemispheres were microdissected and separated into cortical and hippocampal tissues, then flash‐frozen for biochemical analysis. The samples were pulverized using a Bessman Tissue Pulverizer kit, and half of pulverized tissue was homogenized in 1000 μL/150 mg (cortex) or 150 μL (hippocampus) Tissue Protein Extraction Reagent (TPER; Life Technologies, Grand Island, NY, USA) containing protease (Roche, Indianapolis, IN, USA) and phosphatase inhibitors (Sigma‐Aldrich) before centrifugation at 100,000 × *g* for 1 h at 4°C to generate TPER‐soluble fractions. For formic‐acid fractions, pellets from the TPER‐soluble fraction were homogenized in 500 μL (cortex) or 75 μL (hippocampus) 70% formic acid followed by centrifugation at 100,000 × *g* for 1 h at 4°C. Protein concentration was calculated via Bradford Protein Assay. Human Aβ in soluble and insoluble fractions was quantified using the V‐PLEX Aβ Peptide Panel 1 (6E10) (K15200G‐1; Meso Scale Discovery, Rockville, MD, USA) and the neurofilament light chain (NfL) in plasma and cortex was quantified using the R‐Plex Human Neurofilament L Assay (K1517XR‐2; Meso Scale Discovery), all according to the manufacturer's instructions.

### 2.6 RNA sequencing analysis

#### 2.6.1 Library preparation

RNA‐seq was performed on microdissected cortical and hippocampal brain regions at 4 months and on the hippocampus at 12 months. Total RNA was extracted using RNeasy Mini Kit (Qiagen) on a QIAcube (Qiagen) liquid handling platform as per the manufacturer's instructions. Only samples with an RNA integrity number (RIN) ≥7.0, measured via Qubit RNA IQ Assay (Invitrogen), were used for cDNA synthesis. Barcodes and adapters were added, and subsequent steps were performed following cDNA synthesis and amplification using the Smart‐seq2^36^ standard protocol. The DNA Prep Kit (Illumina) using the epMotion 5070 TMX (Eppendorf) automated pipetting system was used to generate libraries from these brain regions, which were base‐pair selected based on Agilent 2100 Bioanalyzer profiles and normalized by a KAPA Library Quantification Kit (Roche). All the libraries were sequenced using paired‐end 43‐bp mode on the Illumina NextSeq500 platform with >14 million reads per sample.

#### 2.6.2 Custom reference genome construction

Because no annotated reference of the eight CC founder strains was readily available, the total transcriptomes from each of the eight (A/J, C57BL/6J, 129S1Sv/ImJ, NOD/ShiLtJ, NZO/H1LtJ, CAST/EiJ, PWK/PhJ, and WSB/EiJ) founder inbred strains were used in conjunction with the haplotype reconstructions based on dense genotyping of the most recent common ancestors (MRCAs) of the CC strains, to generate a custom CC line reference transcriptome. Dense genotyping was performed by JAX. The haplotype files are available at https://csbio.unc.edu/CCstatus/CCGenomes/. The haplotype file contained chromosomal information about the start and end position of the founder strain and the transcriptomes included in that region, which were extracted using the total transcriptomes of the founder strains. Since the mice were F1 progeny of C57BL/6J females crossed with different CC males, the transcriptomes of the two were combined to generate a custom transcriptome for each of the F1 strains.

#### 2.6.3 Alignment

The paired‐end raw reads from the sequencing were mapped to the custom transcriptome using Bowtie (version 1.2.3) and Samtools (version 1.15.1). Subsequently, Emase (version 0.10.16) was used for expected gene quantification for uniquely aligned reads. The transcripts per million (TPM) quants generated were also assessed for effects from biological variables (mouse line, genotype, age, sex) and technical covariates (batch, sequencing biases, gene‐expression principal components [PCs]) related to sequencing quality, which were computed using PicardTools (version 2.1.1). Low expressed genes (defined as not expressed in 80% of the samples) were removed according to standard practices for RNA‐seq experiments to reduce noise. To remove the effects of the covariates mentioned above, a linear regression model was implemented as follows:

$$\begin{array}{ccc} & & \mathrm{lm}\left(\mathrm{expression}∼\mathrm{line}+\mathrm{genotype}+\mathrm{sex}+\mathrm{age}+\mathrm{Seq}.\mathrm{PC}1+\mathrm{Seq}\right.\\ & & \;\left..\mathrm{PC}2+\mathrm{Seq}.\mathrm{PC}3+\mathrm{Seq}.\mathrm{PC}4+\mathrm{Seq}.\mathrm{PC}5\right), \end{array}$$

where Seq.PCs are sequencing PCs obtained from aggregating sequencing metrics obtained from Picard tools.

#### 2.6.4 Differential gene expression

Differential gene expression analysis was performed using edgeR (version 3.38.4). Principal component (PC) analysis of the normalized gene expression data was performed to understand the biological and technical covariates affecting the data. Following this, the samples were separated by gender to assess sex‐related variations. Heatmaps depicting the number of differentially expressed genes (DEGs) between the WT F1 progenies of the different crosses were plotted using the ggplot2 (version 3.3.6) package. Genes of interest were selected based on log2(Fold Change) > 1 and false discovery rate (FDR) <0.05. Gene ontology (GO) enrichment was performed using the enrichR (version 3.1) package in R (version 4.2.1). Volcano plots for visualizing differences between WT and 5xFAD samples were made using the EnhancedVolcano (version 1.14.0) package. Venn diagrams for DEGs were constructed using VennDiagram package (version 1.6.20).^37^

#### 2.6.5 Accelerating medicines project‐Alzheimer's disease (AMP‐AD) module enrichment

The gene list generated from the differential analysis comparing the WT F1 progeny of the CC and B6J to their 5xFAD counterparts within each line was separated based on gene directionality (up‐ or downregulated, ie, log2(Fold Change) > 1 and log2(Fold Change) < −1) and significance (FDR < 0.05 for both). These lists were then enriched for the 30 AMP‐AD modules^38^ using a two‐sided Fisher's exact test with 95% confidence intervals with the help of the “fisher.test” function in R. The *p* value was computed using the number of genes overlapping between our gene lists and those included in the human AMP‐AD modules.

#### 2.6.6 Consensus weighted correlation gene network analysis

To identify mRNA modules that are conserved across various transgenic mouse strains, we used a consensus co‐expression network analysis (cWGCNA) approach using the WGCNA package in R. A signed consensus network for each brain region was created individually to help identify the common expression patterns across genetic backgrounds. Our goal was to investigate mouse brain co‐expression networks that were age‐ and sex‐specific but independent of genetic backgrounds. A consensus network analysis approach provided the groups of co‐expressed genes (or modules) that were not affected by mouse genetic background. In brief, a signed similarity matrix was generated after calculating bi‐weighted mid‐correlations for all pairs of genes. The expression profiles were assigned a signed value based on the similarities between the genes depicted in the signed networks. To further emphasize strong correlations and minimize the effect of weak correlation (an exponential scale), this signed similarity matrix was then also raised to power β, which resulted in an adjacency matrix and later transformed into a topological overlap matrix. Following published methods,^39^, ^40^ co‐expression patterns conserved across different mouse strains were identified by generating this consensus network. Each individual network generated was scaled (consensus scaling quantile = 0.2) and a threshold was selected based on a scale‐free *R* ^2^ fit of 0.8 (softpower = 16). For this topological overlap (TO) component‐wise minimum values were calculated to create a consensus network, which was then further used as a distance measure 1 − TO (dissTOM) to cluster the genes hierarchically. A standard dynamic tree‐cutting algorithm was applied to generate initial module assignments (cutreeHybrid, using default parameters except deepSplit = 4, cutHeight = 0.999, minModulesize = 100, dthresh = 0.25, and pamStage = FALSE). The resulting modules or groups of co‐expressed genes were used to calculate module eigengenes (MEs; or the first PC of the module). Modules were annotated for top GO terms using the GOElite (version 1.2.5) and enrichR package, and primary ontology was assigned based on highest *z*‐score. We performed module preservation analysis using mRNA module definitions. Correlations were performed to extract age‐ or sex‐specific modules between the MEs generated and different biological and technical traits (eg, sex, age). Module hubs were defined by calculating module membership (kME) values, which are the Pearson correlations between each gene and each ME. A threshold of kME < 0.7 was used to remove genes from the module. Network visualization was done using the iGraph package in R.

A similar cWGCNA was performed separately to look for genotypic variations in the dataset in the hippocampus using the WGCNA package in R as described above. A thresholding power of 14 was selected. Again, the new module assignments were determined using a dynamic tree‐cutting algorithm (cutreeHybrid, using default parameters except deepSplit = 4, cutHeight = 0.999, minModulesize = 100, dthresh = 0.2, and pamStage = FALSE). Downstream processing was performed as described for co‐expression analysis. Module definitions from the network analysis were used to create synthetic eigengenes and were used to understand the trajectory of various modules across time points as well as to look at the genotype‐specific and sex‐specific MEs.

#### 2.6.7 Cell type enrichment

A custom dataset was created using the whole annotated gene list from a previously published mouse brain dataset,^41^ which includes the top 100 cell type‐specific marker genes for five major cell types: neurons, astrocytes, oligodendrocytes, microglia, and endothelial cells. The complete RNA‐seq expression data were used as background for overrepresentation analysis. Next, the Fisher.test function in R was used to perform a two‐sided Fisher's exact test with 95% confidence intervals as described. The *p* values generated from this two‐sided approach for the one‐sided test are equivalent to the hypergeometric *p* values. To run multiple hypergeometric test comparisons these *p* values were further FDR adjusted to correct for false positives.

#### 2.6.8 Gene set PC analysis

Using published work,^14^, ^42^, ^43^, ^44^ we constructed four different annotated gene lists based on their implicated functions: AD‐related, astrocytic, microglia, and neuronal. The expression values of the genes in these lists were plotted across the different CC lines and eigengene values were calculated using the PC analysis for different lists across the lines. The first PC (PC1) are the eigengenes generated, and statistics were run on these values.

### 2.7 Statistics

All the statistical analyses were performed using Prism version 9.0.0 (GraphPad Software, Boston, MA, USA). Amyloid β biochemical and immunohistochemical data were analyzed using a one‐way ANOVA with Dunnett *post hoc* test. For analyses with both WT and 5xFAD groups, two‐way ANOVA with the Dunnett *post hoc* test was used. For all analyses, statistical significance was accepted at *p *< 0.05 and significance expressed as follows: *p < 0.05, **p < 0.01, ***p < 0.001, ****p < 0.0001. 

## 3 RESULTS

### 3.1 Generation of genetically diverse WT‐CC and 5x‐CC lines

**FIGURE 1:**
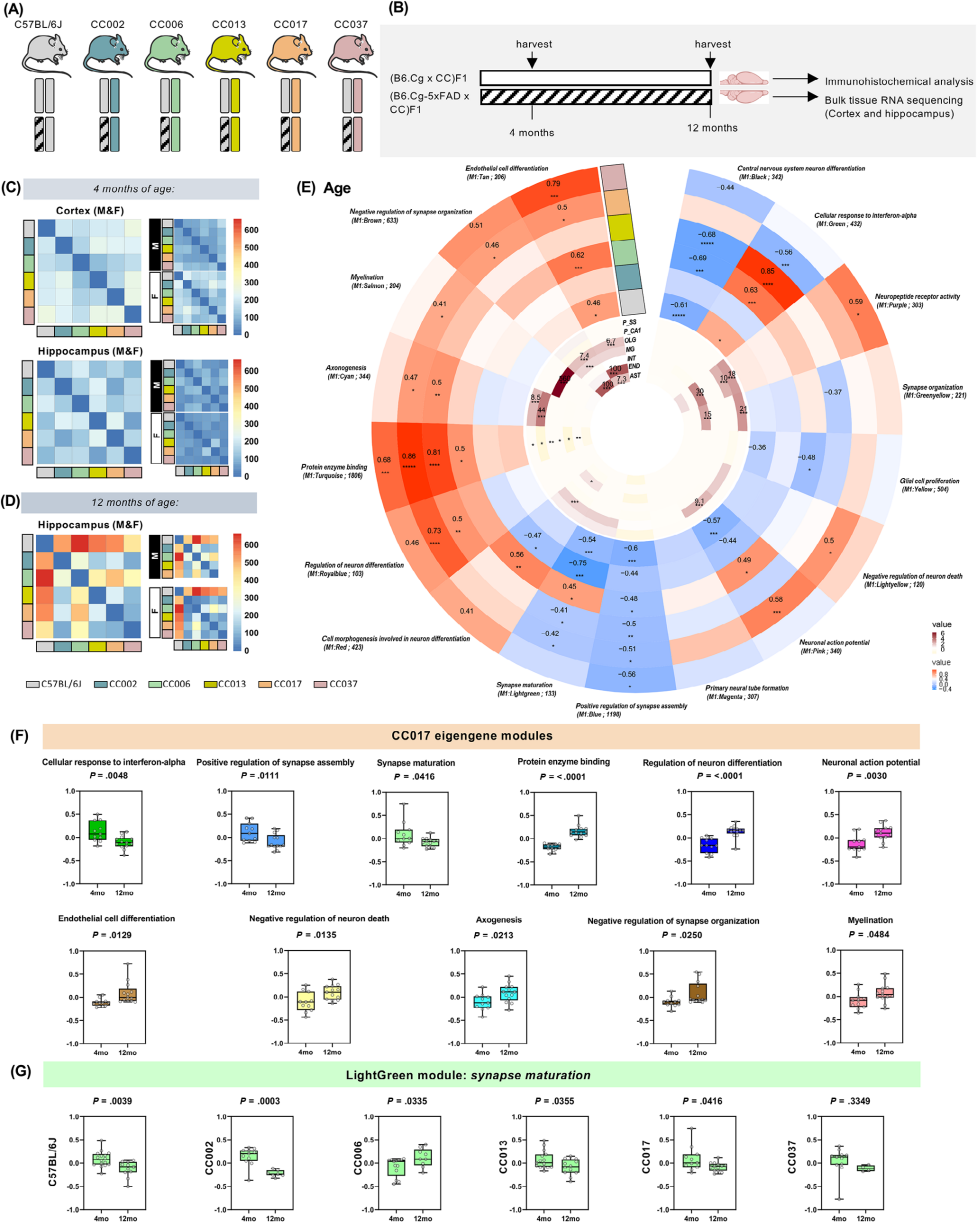
(A) Allelic representation of congenic B6J and (B6J × CCnnn) F1 mice used in this study: female congenic B6J mice hemizygous for the 5xFAD transgene were bred to B6J males, or with males from five different Collaborative Cross (CC) strains (CC002, CC006, CC013, CC017, CC037) to generate genetically diverse F1 offspring. (B) Experimental paradigm: WT‐CC (B6.Cg × CC)F1 and 5x‐CC (B6.Cg‐5xFAD × CC)F1 mice, plus B6.Cg‐5xFAD hemizygous and B6.Cg non‐transgenic mice were sacrificed at 4 and 12 months of age (*n* = 396 total mice) and brains collected for immunohistochemical and bulk RNA‐seq analysis. (C and D) Heatmaps of number of differentially expressed genes (DEGs; FDR < 0.05, log2‐fold change > 1) between the F1 progeny of WT‐CC mice in the cortex and hippocampus at 4 and 12 months. Insets separate animals by sex (F = female, M = male). Blue indicates downregulation and red upregulation in gene expression. Scales are normalized to visualize relative changes across brain regions and time points. The identity of the male in each cross is indicated below panel D. (E) Circular heatmap depicting the consensus‐weighted gene co‐expression network built using cWGCNA, which was used to explore age‐related changes conserved across the WT‐CC lines in the hippocampus, consisting of 17 gene co‐expression modules. GO‐Elite pathway analysis was performed to identify biological processes represented by each module (outer circle; red represents a positive correlation, blue a negative correlation; significant correlation of 0.1 and −0.1; FDR < 0.05). Cell type enrichment analysis was assessed on each module via overlap with cell type‐specific gene lists of pyramidal neurons in the somatosensory cortex (P_SS), pyramidal neurons in the CA1 (P_CA1), oligodendrocytes (OLG), microglia (MG), interneurons (INT), endothelial cells (END), and astrocytes (AST) (inner circle; dark maroon symbolizes high enrichment, pale yellow indicates no enrichment; enrichment threshold > 0.6). (F) Module eigengene levels by age for the 11 significantly trait‐correlated modules in WT‐CC017 mice (*n* = 11 for 4 months, *n* = 13 for 12 months). Based on their trajectories, modules are classified as downregulated (M1: Green – cellular response to interferon‐alpha, M1: Blue – positive regulation of synapse assembly, M1: LightGreen – synapse maturation) or upregulated (M1: Turquoise – protein enzyme binding, M1: RoyalBlue – regulation of neuron differentiation, M1: Pink – neuronal action potential, M1: Tan – endothelial cell differentiation, M1: LightYellow – negative regulation of neuron death, M1: Cyan – axonogenesis, M1: Brown – negative regulation of synapse organization, M1: Salmon – myelination). (G) Module eigengene plots for M1: LightGreen module shown across WT‐CC lines (*n* = 16 for 4‐month‐old WT‐C57BL/6J, *n* = 11 for 12‐month‐old WT‐C57BL/6J; *n* = 11 for 4‐month‐old WT‐CC002, *n* = 8 for 12‐month‐old WT‐CC002; *n* = 13 for 4‐month‐old WT‐CC006, *n* = 9 for 12‐month‐old WT‐CC006; *n* = 15 for 4‐month‐old WT‐CC013, *n* = 11 for 12‐month‐old WT‐CC013; *n* = 11 for 4‐month‐old WT‐CC017, *n* = 13 for 12‐month‐old WT‐CC017; *n* = 11 for 4‐month‐old WT‐CC037, *n* = 5 for 12‐month‐old WT‐CC037). Differences in module eigengene by age were assessed by Fisher's test. Statistical significance is denoted by *p < 0.05, **p < 0.01, ***p < 0.001, ****p < 0.0001.

To investigate phenotypic and transcriptional differences due to genetic diversity, we crossed female 5xFAD hemizygous congenic C57BL/6J (B6) mice to males from five different CC lines to generate genetically diverse mice with (B6.Cg‐5xFAD X CC)F1 and without (B6.Cg x CC)F1 FAD mutations (Figure 1A). We also crossed B6.Cg‐5xFAD female mice with B6 male mice to generate congenic B6 non‐transgenic and 5xFAD hemizygous mice as controls. Mouse brains were collected for immunohistochemical and transcriptional analysis at 4 and 12 months of age (Figure 1B). With this breeding scheme, the mitochondrial DNA of all F1 mice is from the B6 background. Hereafter, for simplicity we refer to the non‐transgenic (B6J.Cg x B6)F1 mice as WT and the corresponding (B6J.Cg x CC)F1 and (B6J.Cg‐5xFAD x CC)F1 mice as WT‐CC and 5x‐CC, respectively (eg, WT‐CC002, 5x‐CC002).

### 3.2 Transcriptional diversity is maintained across age and is not sex‐dependent in WT‐CC mice

To ascertain the (non‐transgenic‐mediated) transcriptomic changes between the different WT‐CC F1 strains, we performed RNA‐seq and aligned it to a custom CC line reference using the total transcriptomes of the eight founder strains as no reference transcriptome was available. We calculated the total number of DEGs between the WT‐CC mice (Figure 1C‐D) using DEGs (FDR < 0.05, log2‐fold change > 1) as a proxy to detect strain divergence. At 12 months of age, WT mice had the highest number of DEGs compared to WT‐CC mice, followed by WT‐CC006, indicating that congenic B6J mice were the most divergent strain (Figure 1D). To further investigate whether this disparity was driven by sex, we separated the cohort by sex, re‐analyzed the dataset, and observed that at 12 months of age the B6J strain displayed the highest number of DEGs, in both sexes, indicating that these genetic changes were not impacted by sex (Figure 1D). We next sought to examine the potential of WT‐CCs in identifying genetic changes associated with specific biological processes, including those associated with aging. To accomplish this, we used consensus‐weighted gene co‐expression network analysis (cWGCNA), a widely used method of analysis to discover biologically relevant correlation patterns (ie, gene modules) among genes across multiple gene expression datasets. Consensus WGCNA was performed on the six different lines – WT‐B6J, WT‐CC002, WT‐CC006, WT‐CC013, WT‐CC017, and WT‐CC037 – to examine the age‐related changes conserved across the different strains in hippocampal brain regions.^39^ We identified 17 consensus modules significantly correlated with age – three negatively (M1: Blue, M1: Yellow, M1: Black) and eight positively correlated (M1: Tan, M1: Brown, M1: Salmon, M1: Cyan, M1: Turquoise, M1: RoyalBlue, M1: LightYellow, M1: Purple; FDR < 0.05; Figure 1E).

To better understand the modules, we also performed GO and cell type‐specific marker enrichment analysis to gain insight into each module for functional pathway and cell type‐specific information (Figure 1E). As expected, the *myelination* module is enriched for oligodendrocytes (OLG) and the *neuropeptide receptor activity* module enriched for interneurons (INT) as well as CA1 (P_CA1) and somatosensory cortex pyramidal neurons (P_SS) (Figure 1E). We found that *protein enzyme binding* and *endothelial cell differentiation* is upregulated with aging in most of the lines, whereas *positive regulation of synapse assembly* and *synapse maturation* is downregulated with aging (Figure 1E). Interestingly, all WT‐CC lines are negatively correlated to the M1: Lightgreen module, except for WT‐CC006, which is positively correlated with this module, indicating a differential response to aging in genes associated with *synapse maturation*. Strikingly, a large fraction of the modules is significantly correlated to WT‐CC017, indicating that this strain exhibits unique genetic changes associated with specific pathways, including *myelination*, *negative regulation of synapse organization*, and *negative regulation of neuronal death* (Figure 1E). Across different time points, WT‐CC017 display distinct trajectories of each module during aging, including a downregulation in *cellular response to interferon‐alpha* (Green) (Figure 1F). In the *synapse maturation* module (LightGreen), which is significantly regulated with respect to age and across the lines, we observe that all strains except for WT‐CC013 exhibit a downregulation in the genes associated with *synapse maturation* (Figure 1G). We also performed cWGCNA to detect sex‐related changes but found no significant correlation with sex across the WT‐CC lines, except for a negative correlation with the M1: Blue and M1: Magenta modules in WT‐CC037 mice and the M1: Purple and M1: Greenyellow in WT‐C57BL/6J mice (Figure S1). These modules are associated with *positive regulation of synapse assembly* (M1: Blue), *primary neural tube formation* (M1: Magenta), *synapse organization* (M1: Greenyellow), and *neuropeptide receptor activity* (M1: Purple) (Figure S1). Our analysis of the WT‐CC progeny, comprising approximately 400 brains, demonstrates the utility of this small panel to quantify genetic variation between different background strains, as well as to construct a highly powered robust gene co‐expression network. Here, introducing genetic diversity via the WT‐CC F1 panel shows genetic diversity is maintained during aging, as seen by a replicated pattern of alterations correlated to age across the different genetic lines, which is not sex driven.

### 3.3 Introduction of genetic diversity to 5xFAD model leads to robust reductions in plaque and Aβ deposition

**FIGURE 2:**
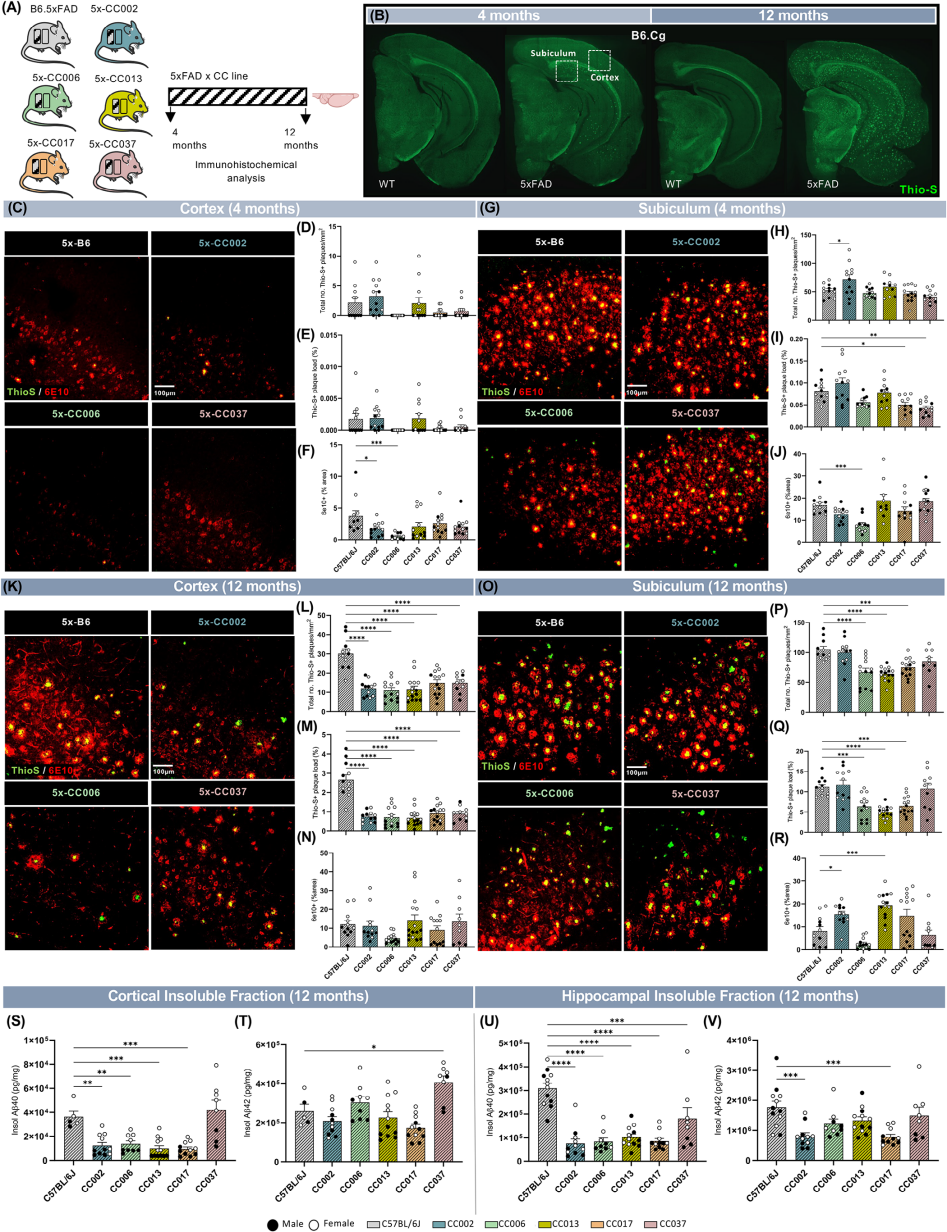
(A) Graphical representation of B6J.Cg‐5xFAD (5xFAD) and (B6J.Cg‐5xFAD × CCnnn)F1 (5x‐CCnnn) mice that underwent immunohistochemical analysis for Aβ‐related pathology. (B) Representative whole‐brain images of 4‐ and 12‐month‐old WT non‐transgenic (B6J.Cg) and 5xFAD (B6J.Cg‐5x‐FAD hemizygous) mice stained for dense‐core plaques using Thioflavin‐S (ThioS, green). (C, G) Representative confocal images of the somatosensory cortex (C) and subiculum (G) in 4‐month‐old 5x‐B6J and select 5x‐CC lines (5x‐CC002, 5x‐CC006, and 5x‐CC037) immunolabeled for ThioS (green) and 6E10 (red). (D–F, H–J) Quantification of plaque load in cortex (D–F) and subiculum (H–J) of 4‐month‐old 5x‐B6J and select 5x‐CC lines, as assessed by density of ThioS^+^ plaques (D, H), ThioS^+^ % area (E, I), and 6E10^+^ % area (F, J) per field of view. (K, O) Representative confocal images of somatosensory cortex (K) and subiculum (O) in 12‐month‐old 5x‐B6J and select 5x‐CC lines immunolabeled for ThioS (green) and 6E10 (red). (L–N, P–R) Quantification of plaque load in the cortex (L–N) and subiculum (P–R) of 12‐month‐old 5x‐B6J and select 5x‐CC lines, as assessed by density of ThioS^+^ plaques (L, P), ThioS^+^ % area (M, Q), and 6E10^+^ % area (N, R) per field of view. Scale bar = 100 μm. (S–V) Quantification of insoluble Aβ40 and Aβ42 protein in the cortex (S–T) and hippocampus (U–V). Data are represented as mean ± SEM. Statistical analysis was performed using a one‐way ANOVA with Dunnett test. *p < 0.05, **p < 0.01, ***p < 0.001, ****p < 0.0001.

To investigate the impact of genetic diversity on amyloid plaque pathology, we performed immunostaining for ThioS and 6E10, markers for amyloid plaques and Aβ, in the brains of 4‐ and 12‐month‐old genetically diverse F1 mice generated from crossing males from the five different CC lines to B6.Cg‐5xFAD hemizygous females (ie, 5xCC), as well as age‐matched B6.Cg‐5xFAD mice (Figure 2A). As previous studies reported a spatially and temporally defined pattern of plaque formation in specific neocortical and hippocampal regions, we analyzed the plaque load in both the cortex and subiculum, a region of the hippocampus where plaques first appear in 5xFAD mice.^45^ Representative images for WT and 5xFAD hemizygotes on the congenic C57BL/6J background strain (B6.Cg‐5xFAD) demonstrated a significant plaque load in 5xFAD hemizygous animals at both 4‐ and 12‐month time points that was absent in the non‐transgenic WT littermate mice (Figure 2B). Confocal microscopy and image quantification analysis revealed significant differences in plaque and parenchymal Aβ deposition between the different 5x‐CC F1 animals (Figure 2C‐R). At 4 months of age, there was a significant reduction in Aβ accumulation in 5x‐CC006 compared to 5x‐B6J in the cortex and subiculum (Figure 2C, F, G, J), as well as a reduction in amyloid plaque load in 5x‐CC017 and 5x‐CC037 compared to 5x‐B6J in the subiculum (Figure 2G, I). We also observed a significant elevation in the density of ThioS^+^ amyloid plaques in 5x‐CC002 compared to 5x‐B6J (Figure 2G, H). At 12 months, 5x‐B6J mice have the greatest amyloid plaque load compared to all 5x‐CC lines (Figure 2K‐Q), with all 5x‐CC lines showing a significant reduction in the total number of ThioS^+^ amyloid plaques and plaque load compared to 5x‐B6J in the cortex (Figure 2K ‐M, Figure S2A). In the subiculum, we observed a significant decrease in the total number of ThioS^+^ amyloid plaques and plaque load in 5x‐CC006, 5x‐CC013, and 5x‐CC017 compared to 5x‐B6J (Figure 2O‐Q, Figure S2B). We also observed a significant increase in Aβ deposition in 5x‐CC002 and 5x‐CC013 compared to 5x‐B6J in the subiculum (Figure 2O, R, Figure S2B). Transgene expression was also assessed, with no significant change observed in *Thy1* across the 5x‐CC lines (Figure S2C).

To further investigate the effect of different genetic backgrounds on amyloidosis, we measured Aβ40 and Aβ42 levels in detergent soluble and insoluble fractions from microdissected cortices and hippocampi. In the cortex of 4‐month‐old animals, we observed a significant increase in soluble Aβ40 in 5x‐CC006 and 5x‐CC037 and soluble Aβ42 in 5x‐CC002 compared to 5x‐B6J (Figure S2D‐E). Consistent with histology, 5x‐CC006 has lower insoluble Aβ40 and Aβ42 in the hippocampus, along with 5x‐CC037, compared to 5x‐B6J (Figure S2J‐K). At 12 months of age, and consistent with reduced plaque deposition in these mice, we observed lower soluble and insoluble Aβ40 in 5x‐CC002, 5x‐CC006, 5x‐CC013, and 5x‐CC017 compared to 5x‐B6J in cortical extracts (Figure 2S, Figure S2L), as well as a significant reduction in insoluble Aβ40 in all 5x‐CC lines compared to 5x‐B6J in the hippocampus (Figure 2U). We also observed less insoluble Aβ42 in 5x‐CC002 and 5x‐CC017 compared to 5x‐B6J in hippocampal fractions (Figure 2V). Despite this, we also observed a significant elevation in insoluble and soluble Aβ42 in 5x‐CC037 and 5x‐CC002 compared to 5x‐B6J in cortical and hippocampal extracts, respectively (Figure 2T, Figure S2O). Taken together, these data indicated that the congenic C57BL/6J (B6J) genetic background exhibited the most susceptibility to plaque development and Aβ accumulation in response to the 5xFAD transgene compared to the B6J x CC F1 strains analyzed. We also identified 5x‐CC006 mice as having a genetic makeup that was relatively resilient to plaque pathology, as evidenced by decreased ThioS^+^ amyloid and 6E10^+^ Aβ immunostaining and reduced levels of insoluble Aβ levels compared to 5x‐B6J mice.

### 3.4 Microglial densities mirror the alterations in plaque loads across the 5x‐CC lines

**FIGURE 3:**
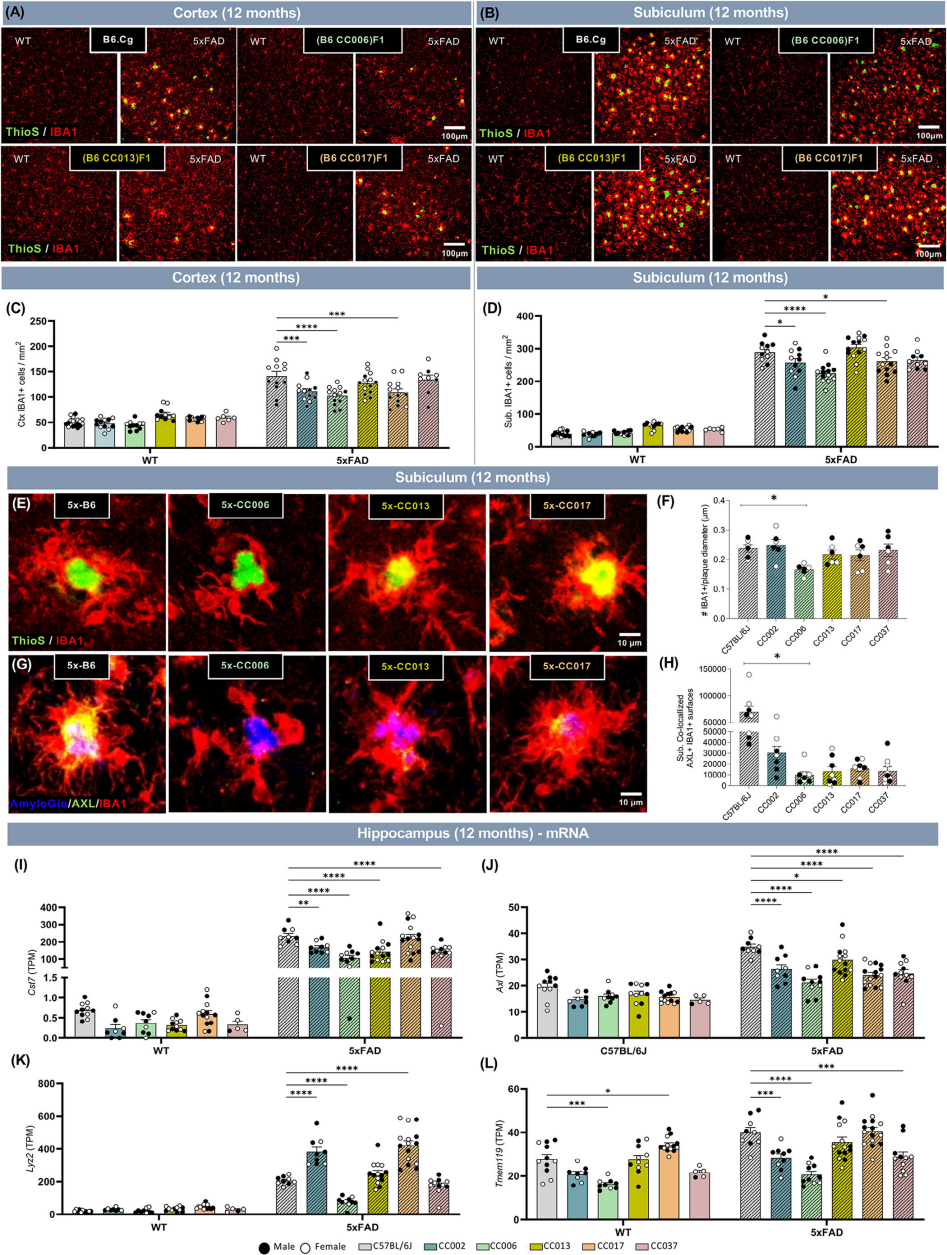
(A and B) Representative confocal images of the somatosensory cortex (A) and subiculum (B) in select 12‐month‐old non‐transgenic (WT) and 5xFAD transgene carrying B6.Cg, or F1 progeny of B6J.Cg × Collaborative Cross (CC) mice (WT‐B6J, 5x‐B6J, WT‐CC006, 5x‐CC006, WT‐CC013, 5x‐CC013, WT‐CC017, and 5x‐CC017) immunolabeled for Thioflavin‐S (ThioS, green) and microglia (IBA1, red). Scale bar = 100 μm. (C and D) Quantification of microglia per square millimeter in the cortex (C) and subiculum (D) at 12 months. (E) Higher‐resolution representative images of plaque‐associated microglia in subiculum of select 12‐month‐old 5x‐CC lines stained for plaques (ThioS, green) and microglia (IBA1 (red). (F) Quantification of average number of microglia surrounding an amyloid plaque. (G) Higher‐resolution representative images of plaque‐associated microglia in subiculum of select 12‐month‐old 5x‐CC lines stained for plaques (AmyloGlo, blue), microglia (IBA1 (red), and AXL (green, a marker for microglial activation). (H) Quantification of amount of microglial AXL (co‐localized IBA1+AXL+) staining. (I and J) Expression of disease‐associated microglia (DAM) and homeostatic microglia genes quantified by number of transcripts per million reads (TPM) in 12‐month‐old WT‐CC and 5x‐CC mice in hippocampus (*n* = 125 mice across 10 lines). Data are represented as mean ± SEM. Statistical analysis was performed using a two‐way ANOVA with Dunnett test. *p < 0.05, **p < 0.01, ***p < 0.001, ****p < 0.0001.

Microglia, the primary immune cells of the CNS, surround amyloid plaques during AD and have been implicated in disease pathogenesis.^46^ Given the key role of microglia in Aβ‐related pathology and the significant differences in Aβ plaque deposition between the different 5x‐CC strains, we next stained mouse brains for ThioS and IBA1, a microglial cell marker, and evaluated microglial density in both WT‐ and 5x‐CC strains at 4 and 12 months of age to characterize the impact of genetic background on this CNS immune cell type (Figure 3A‐F; Figure S3A‐D). Genetic background has a significant effect on microglial cell numbers, as seen by a significant main effect of strain in the cortex (F [5, 132] = 7.609; *p* < 0.0001; Figure S3C) and in the subiculum (F [5, 131] = 6.673; *p* < 0.0001; Figure S3D) in 4‐month‐old mice (Figure S3A‐B), which is also present in the cortex (F [5, 131] = 7.266; *p* < 0.0001; Figure 3C) and subiculum (F [5, 130] = 11.23; *p* < 0.0001; Figure 3D) at 12 months of age. We also observed a significant main effect of genotype on microglial numbers (F [1, 132] = 13.87; *p* = 0.0003; Figure S3C) in the cortex and (F [1, 131] = 501.7; *p* < 0.0001; Figure S3D) in the subiculum at 4 months (Figure S3A‐B), as well as the cortex (F [1, 131] = 452.6; *p* < 0.0001; Figure 3C) and subiculum (F [1, 130] = 2565; *p* < 0.0001; Figure 3D) at 12 months, indicating that, as expected, numbers of microglia are also impacted by the presence of the 5xFAD transgene. Between strains, we observed no significant difference between microglial numbers in WT‐CC mice compared to WT‐B6J mice (Figure 3C‐D, Figure S3C‐D). In 5x‐CC strains, there were fewer microglia in the subiculum of 4‐month‐old 5x‐CC006 compared to 5x‐B6J mice (Figure S3D), which could reflect this mouse strain's decreased hippocampal Aβ. Interestingly, we also observed more microglia in 5x‐CC037 in the subiculum at 4 months (Figure S3D), although these mice displayed fewer hippocampal plaques and lower insoluble Aβ. This is the only line in which we did not observe fewer microglia in the presence of less pathology. These data could implicate a differential microglial response to plaques in the 5x‐CC037 strain in the early stages of plaque development. In line with our Aβ histological and biochemical data, in which we observed fewer ThioS^+^ plaques and lower insoluble Aβ, numbers of microglia were significantly decreased in several 5x‐CC lines compared to 5x‐B6J mice, including 5x‐CC002, 5x‐CC006, 5x‐CC0017 in the cortex and subiculum at 12 months of age (Figure 3A‐D).

Next, we assessed the effect of genetic diversity on microglial activation and response to plaques. First, we quantified the number of IBA1+ cells surrounding ThioS+ plaques. These data revealed that 5x‐CC006 mice had a diminished microglial response, as indicated by fewer microglia surrounding plaques compared to 5x‐B6J mice (Figure 3E‐F). We next stained mouse brains for AXL, a marker for microglial activation, and observed a significant decrease in the proportion of AXL+ microglia in the subiculum at 12 months of age (Figure 3G‐H, Figure S3E). Accompanying these were decreases in the mRNA expression of microglial activation, disease‐associated microglia (DAM), and AD‐related genes such as *Cst7*, *Axl*, *Trem2*, *Clu*, and *Abca7* in several 5x‐CC lines (5x‐CC002, 5x‐CC006, 5x‐CC013, 5x‐CC017) compared to 5x‐B6J (Figure 3I‐J, Figure S3F‐H). We also observed significant decreases in the homeostatic microglial gene *Tmem119* in 5x‐CC002, 5x‐CC006, and 5x‐CC037 compared to 5x‐B6J (Figure 3L). Interestingly, we observed a significant decrease in the expression of *Lyz2* in 5x‐CC006 but a significant elevation in the expression in 5x‐CC002 and 5x‐CC017 compared to 5x‐B6J mice (Figure 3K). 5x‐CC006 also expressed less *Apoe* while 5x‐CC013 expressed more *Apoe* compared to 5x‐B6J mice (Figure S3I). Together, these results provide evidence that genetic background can impact the microglial response in the presence of 5xFAD transgene (ie, amyloid‐related AD pathology) and that for the majority of 5x‐CC lines the microglial response or DAM signature appears diminished, particularly 5x‐CC006, compared to 5x‐B6J, similar to their reduction in plaque pathology.

### 3.5 WT and 5x‐CC strains exhibit profound alterations in astrocytes

**FIGURE 4:**
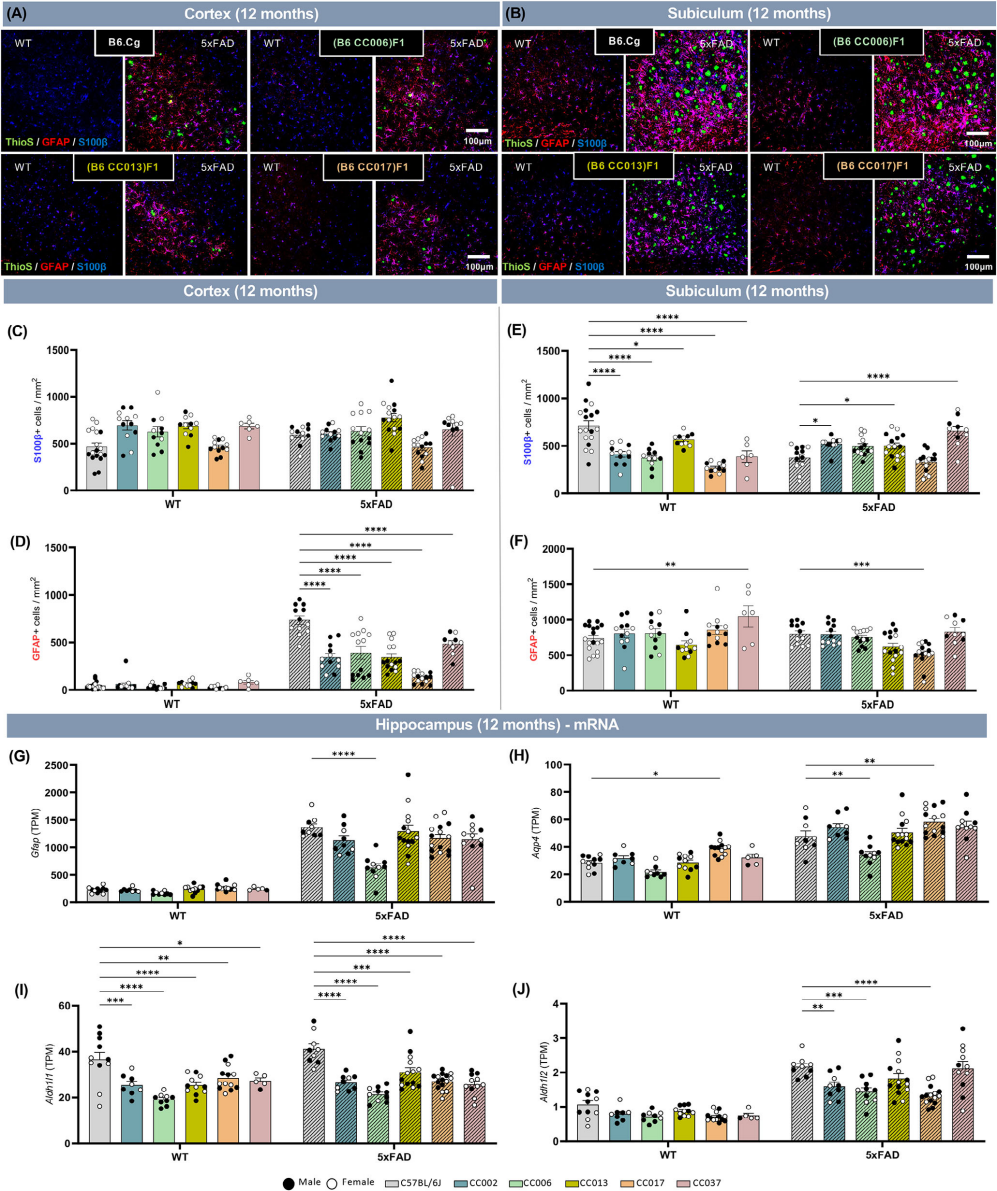
(A and B) Representative confocal images of somatosensory cortex (A) and subiculum (B) in select 12‐month‐old non‐transgenic (WT) and 5xFAD transgene carrying B6J.Cg, or F1 progeny of B6J.Cg × CC mice (WT‐B6J, 5x‐B6J, WT‐CC006, 5x‐CC006, WT‐CC013, 5x‐CC013, WT‐CC017, and 5x‐CC017) immunolabeled for Thioflavin‐S (ThioS, green), reactive astrocytes (GFAP, red), and all astrocytes (S100β, blue). Scale bar = 100 μm. (C–F) Quantification of S100β^+^ (C, E) and GFAP^+^ (D, F) astrocytes per square millimeter in the cortex (C and D) and subiculum (E and F). (G–J) Expression of astrocyte‐related genes quantified by number of transcripts per million reads (TPM) in 12‐month‐old WT‐CC and 5x‐CC mice in hippocampus (*n* = 125 mice across 10 lines). Data are represented as mean ± SEM. Statistical analysis was performed using a two‐way ANOVA with Dunnett test. *p < 0.05, **p < 0.01, ***p < 0.001, ****p < 0.0001.

In addition to microglia, the glial response to amyloid plaques in AD also appears to be mediated by astrocytes.^47^ Thus, we next stained and assessed the influence of genetic diversity on homeostatic and reactive astrocytes using S100β and GFAP, respectively, in mouse brain sections from WT‐CC and 5x‐CC mice at both 4 and 12 months of age (Figure 4A‐B; Figure S4A‐B). At 4 months of age, genetic background significantly impacted the number of S100β^+^ cells in the cortex (F [5, 130] = 12.28; *p* < 0.0001; Figure S4C) and in the subiculum (F [5, 129] = 33.21; *p* < 0.0001; Figure S4E) and, to a lesser extent, GFAP^+^ cells in the cortex (F [5, 128] = 2.311; *p* = 0.0477; Figure S4D) and subiculum (F [5, 129] = 4.805; *p* = 0.0005; Figure S4F). We also showed that genotype (ie, the presence of the 5xFAD transgene) also had a significant main effect on S100β^+^ cells in the cortex (F [1, 130] = 35.05; *p* < 0.0001; Figure S4C) and subiculum (F [1, 129] = 105.3; *p* < 0.0001; Figure S4E) at 4 months, as well as GFAP^+^ cells in the cortex (F [1, 128] = 5.371; *p* = 0.0221; Figure S4D) and subiculum (F [1, 129] = 330.8; *p* < 0.0001; Figure S4F). Between strains, we detected significant elevations in S100β^+^ cells in the cortex in WT‐CC006 and WT‐ CC037 mice compared to WT‐B6J in both the presence and absence of the 5xFAD transgene (Figure S4C). However, in the subiculum, most or all CC‐derived F1 strains – including CC002, CC013, and CC037 – exhibit significantly fewer S100β^+^ cells compared to WT‐B6J (Figure S4E). At 4 months, we detected little to no significant changes in the number of total GFAP^+^ cells between strains, with the exception of a significant reduction in GFAP^+^ cells in the cortex of 5x‐CC017 compared to 5x‐B6J (Figure S4F). At 12 months of age, there was a significant main effect of strain on the number of S100β^+^ cells in the cortex (F [5, 134] = 11.4; *p *< 0.0001; Figure 4A, C) and subiculum (F [5, 135] = 13.76; *p* < 0.0001; Figure 4B, E), as well as the number of GFAP^+^ cells in the cortex (F [5, 133] = 22.43; *p* < 0.0001; Figure 4A, D) and subiculum (F [5, 135] = 5.948; *p* < 0.0001; Figure 4B, F). We also observed a significant main effect of genotype on the number of GFAP^+^ cells in the cortex (F [1, 133] = 332.3; *p* < 0.0001; Figure 4D) and subiculum (F [1, 135] = 9.272; *p* = 0.0028; Figure 4F), but not on the number of S100β^+^ cells (Figure 4A‐E), indicating that reactive but not homeostatic astrocytes are impacted by the presence of the 5xFAD transgene (ie, amyloid pathology). Between strains, we observed a significant reduction in the number of S100β^+^ cells in the subiculum at 12 months of age between all WT‐CC lines and B6J but a significant increase in the number of S100β^+^ cells in 5x‐CC002, 5x‐CC013, and 5x‐CC037 compared to B6J (Figure 4B, E). In the cortex, there is a significant decrease in the number of GFAP^+^ cells at 12 months of age (Figure 4D). In the subiculum, the number of GFAP^+^ cells was significantly increased in WT‐CC037 compared to WT‐B6J mice alongside a significant decrease in GFAP^+^ cells comparing 5x‐CC017 to 5x‐B6J (Figure 4F). In contrast to microglia, in which there was no significant difference between WT‐CCs compared to B6J, we observed profound differences in astrocytes across the WT‐CC lines. For example, WT‐CC006 exhibited a robust increase in cortical S100β^+^ cells in both the absence and presence of the 5xFAD transgene at 4 months of age (Figure S4C). Conversely, 5x‐CC017 exhibited fewer GFAP^+^ cells in the cortex at both time points (Figure 4D, S4D) and in subiculum at 12 months of age (Figure 4F) compared to 5x‐B6J mice, indicative of an altered astrocytic response to plaques. We also analyzed the mRNA level of astrocytic‐related genes and found reduced expression in several 5x‐CC lines compared to 5x‐B6J mice. These included lower expression of *Gfap*, a marker for reactive or disease‐associated astrocytes, and *Aqp4*, a marker for astrocyte endfeet, in 5x‐CC006 compared to 5x‐B6J mice (Figure 4G‐H). We also observed reduced expression of *Aldh1l1* and/or *Aldh1l2*, pan‐astrocyte markers, in several WT‐ and 5x‐CC lines (Figure 4I‐J). Interestingly, we observed a significant elevation in *Aqp4* expression in CC017 mice compared to B6J mice with and without the presence of the 5xFAD transgene (Figure 4H). These data highlight the potential utility of the CC lines to further explore the genetic variants that are associated with (and ultimately responsible for) these specific cellular changes. In 5x‐CC lines, we saw a significant decrease in the number of GFAP^+^ cells in the cortex and expression of astrocyte‐related genes in the hippocampus at 12 months (Figure 4D, G‐J), which aligns with a reduction in IBA1^+^ cells in these brain regions at this time point. Taken together, these findings illustrate the relationship between microglia and reactive astrocytes during AD pathogenesis in the 5xFAD mouse model and indicate that the activation or response of these cells is diminished in 5xFAD hemizygous mice after crossing to the genetically diverse CC lines.

### 3.6 Neurofilament light chain levels indicate increased resistance of 5x‐CC lines to disease progression

**FIGURE 5:**
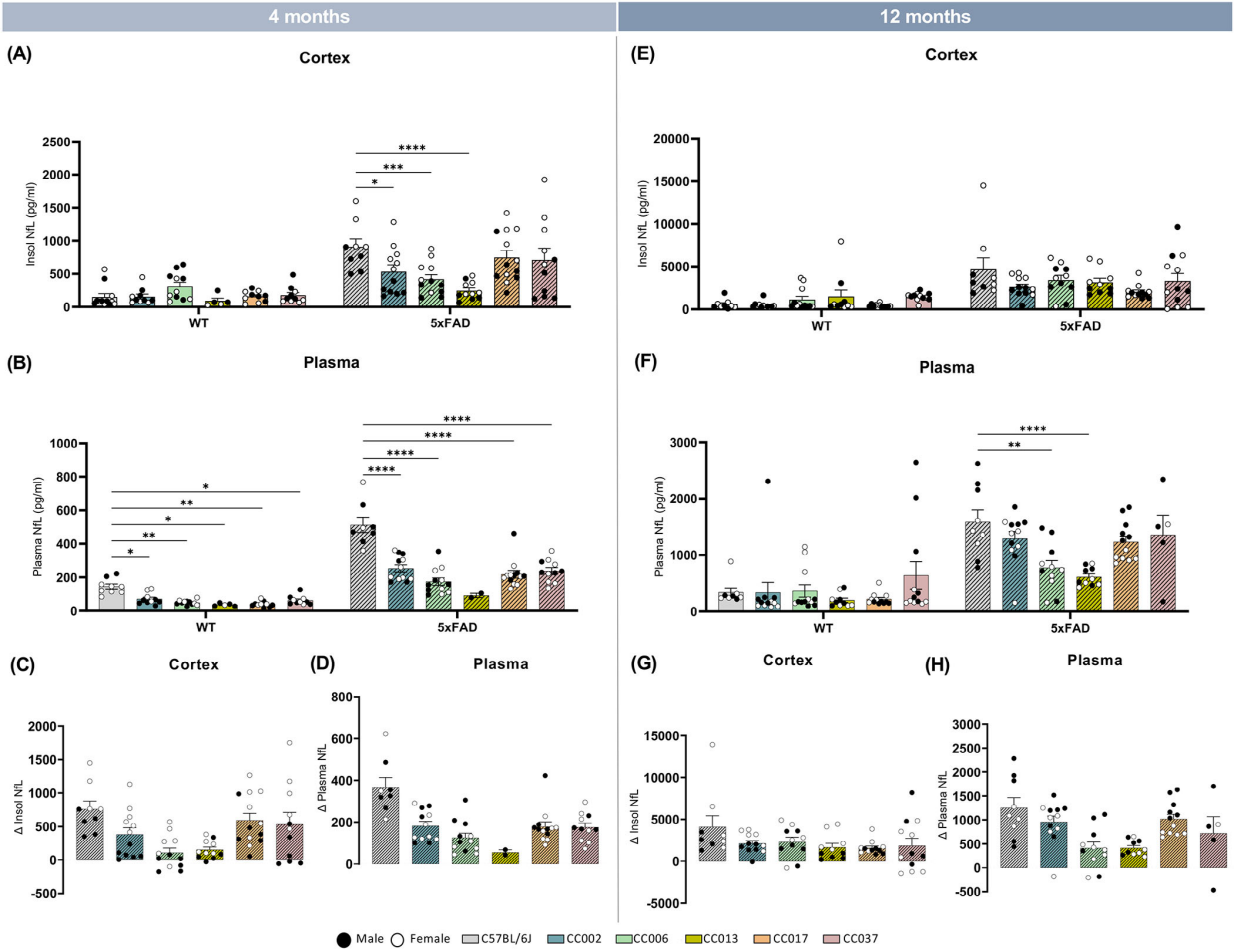
(A–D) Quantification of NfL and ΔNfL in the cortex (A, C, E, G) and plasma (B, D, F, H) of WT‐ and 5x‐CC mice at 4 and 12 months of age. Data are represented as mean ± SEM. Statistical analysis was performed using a two‐way ANOVA with Dunnett test. *p < 0.05, **p < 0.01, ***p < 0.001, ****p < 0.0001.

NfL, a marker for axonal injury/degeneration, has emerged as a potential biomarker for human AD.^48^ Recent studies have also shown that plasma NfL concentration correlates well with plaque load in AD mouse models.^49^, ^50^ To gain insight about the influence of genetic diversity on axonal injury/disease progression, we quantified NfL in both the plasma and cortical insoluble fraction of mice of WT‐ and 5x‐CC strains at 4 and 12 months of age. At 4 months, we detected a significant main effect of background strain and genotype on cortical (strain: F [5, 114] = 3.09; *p* = 0.0118; genotype: (F [1, 114] = 63.84; *p* < 0.0001; Figure 5A, C) and plasma (strain: F [5, 101] = 30.86; *p* < 0.0001; genotype: F [1, 101] = 178.2; *p* < 0.0001; Figure 5B, D) NfL. In the cortex, there was no significant difference between WT‐CCs and WT‐B6J, which was expected given the lack of disease/pathology in this brain region at 4 months of age. However, cortical NfL concentration of 5x‐CC002, 5x‐CC006, and 5x‐CC013 were significantly decreased compared to 5x‐B6J in 4‐month‐old mice (Figure 5A, with differential (Δ) change from WT baseline shown in C). In the plasma, we detected significant and robust reductions in NfL in both WT‐CC and 5x‐CC strains compared to WT‐ and 5x‐B6J, respectively (Figure 5B, with [Δ] change from WT baseline shown in D). At 12 months, genotype (ie, presence of the 5xFAD transgene) but not genetic strain background has a significant effect on NfL concentration in the cortex, as seen by a significant main effect of genotype (F [1, 121] = 54.34; *p* < 0.0001; Figure 5E, with Δ change from WT baseline shown in G). By contrast, plasma NfL concentration is significantly altered by both strain (F [5, 113] = 4.279; *p* = 0.0013) and genotype (F [1, 113] = 82.73; *p* < 0.0001) at 12 months (Figure 5F, with Δ change from WT baseline shown in H). Between strains, we again observe a significant reduction in plasma NfL in 12‐month‐old 5x‐CC006 and 5x‐CC013 compared to 5x‐B6J mice. In line with a reduction in amyloid and Aβ, these data highlight the increased resistance or reduced susceptibility of the CC F1 strains to AD progression, as indicated by reduced NfL levels in these mice compared to 5x‐B6J mice.

### 3.7 Assessment in gene expression overlap between 5x‐CC lines and human AD

**FIGURE 6:**
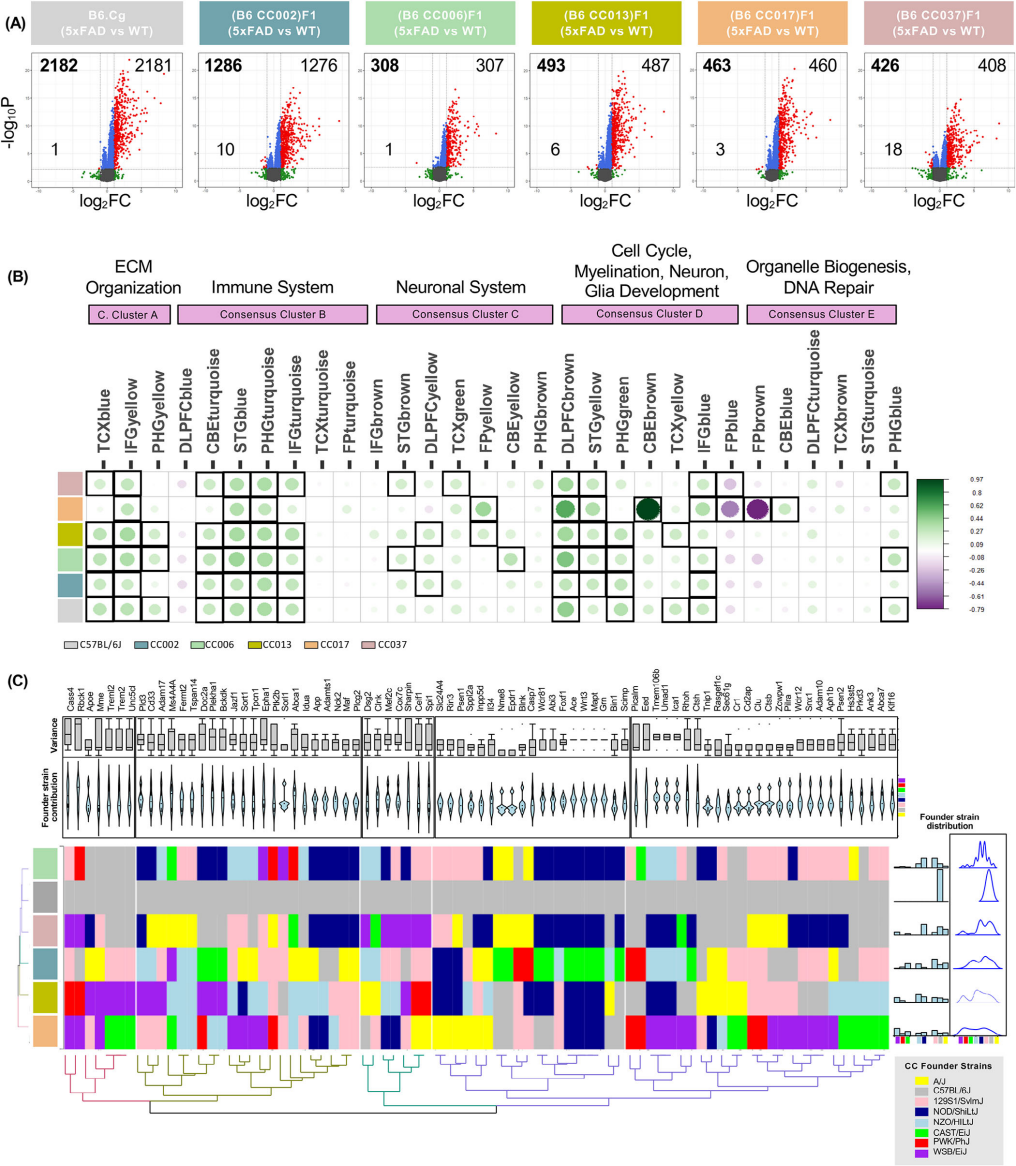
(A) Volcano plots of differentially expressed genes (DEGs; log_2_FC > 1; FDR < 0.05; red dots) in the hippocampus at 12 months of age between 5xFAD‐B6J versus WT‐B6J and 5x‐CC versus their respective WT‐CC counterparts. Gray dots are non‐significant (FDR > 0.05). Green dots indicate |log_2_FC| > 1. Blue dots indicate |log_2_FC| < 1 and FDR < 0.05. The bolded number in the upper left corner represents the total number of DEGs. (B) A correlation analysis between DEGs in 5x‐CC lines and human AD (AMP‐AD) co‐expression modules. Circles within squares with bold borders correspond to significant (*p* < 0.05) positive (green) and negative (purple) Pearson correlation (Pearson correlation ≥ 0.20) coefficients for gene expression changes in 5x‐CC lines associated with human co‐expression modules. Circle size and color intensity are proportional to the correlation. (C) Contribution of founder strain locus information for AD risk genes in each of CC lines visualized as heatmap. Colors denote contribution of each founder strain according to key in bottom right corner of figure. Hierarchical clustering was based on similarity between row (CC lines) and column (AD risk gene loci). Histogram, box, and violin plots were also used to visualize the frequency, distribution, and probability density of founder strain information at AD risk gene loci across the CC lines. (Founder strains: yellow = A/J; gray = C57BL/6J; pink = 129S1/SvlmJ; dark blue = NOD/ShiLtJ; light blue = NZO/HILtJ; green = CAST/EIJ; red = PWK/PhJ; purple = WSB/EiJ.) This analysis was conducted on all animals, including WT and 5xFAD transgene carrying mice across the different genetic backgrounds (*n* = 442 total animals; *n* = 16 for 4‐month‐old WT‐C57BL/6J, *n* = 11 for 12‐month‐old WT‐C57BL/6J; *n* = 11 for 4‐month‐old WT‐CC002, *n* = 8 for 12‐month‐old WT‐CC002; *n* = 13 for 4‐month‐old WT‐CC006, *n* = 9 for 12‐month‐old WT‐CC006; *n* = 15 for 4‐month‐old WT‐CC013, *n* = 11 for 12‐month‐old WT‐CC013; *n* = 11 for 4‐month‐old WT‐CC017, *n* = 13 for 12‐month‐old WT‐CC017; *n* = 11 for 4 mo WT‐CC037, *n* = 5 for 12 mo WT‐CC037; *n* = 11 for 4 mo 5x‐C57BL/6J, *n* = 9 for 12‐month‐old 5x‐C57BL/6J; *n* = 14 for 4‐month‐old 5x‐CC002, *n* = 9 for 12‐month‐old 5x‐CC002; *n* = 18–19 for 4‐month‐old 5x‐CC006, *n* = 10 for 12‐month‐old 5x‐CC006; *n* = 11 for 4‐month‐old 5x‐CC013, *n* = 14 for 12‐month‐old 5x‐CC013; *n* = 13 for 4‐month‐old 5x‐CC017, *n* = 15 for 12‐month‐old 5x‐CC017; *n* = 14 for 4‐month‐old 5x‐CC037, *n* = 11 for 12‐month‐old 5x‐CC037).

Given the differential impact of genetic background strain on AD‐related phenotypes, we analyzed the impact of the 5xFAD transgene on gene expression in genetically diverse strains. RNA‐seq and differential expression analysis were performed comparing 5x‐CC strains to their respective WT‐CC counterparts (plus B6J.Cg‐5xFAD compared to B6J.Cg non‐transgenic) at 12 months of age in hippocampal tissue, that is, the brain region and time point at which we observe the most AD pathological changes. The 5x‐B6J versus congenic WT‐B6J strain comparison had the highest number (2182) of DEGs (Figure 6A) compared to any of the 5x‐B6J CC F1 versus WT B6J CC F1 counterparts, indicating that, of the strains analyzed, the congenic B6J background was the most susceptible to 5xFAD transgene‐induced changes. We also observed that the 5x‐CC006 strain had the fewest (308) DEGs (Figure 6A), which correlates with the 5x‐CC006 line appearing the most resilient to AD‐related processes, displaying the lowest amyloid pathology and numbers of microglia. Next, we explored whether these DEGs were conserved across the strains and found that 239 DEGs were conserved across the strains (Figure S5). These genes include *Trem2, Pycard, Ctss, Ctse, B2m, Cd74, H2‐Ab1, H2‐Aa, H2‐Eb1, Fcgr1, Fcgr2b, C1qa, C3, C1qb, C1qc, Cybb*, and *Tyrobp*, belonging to *complement‐mediated synapse pruning* and *regulation of antigen processing and presentation of peptide antigen via MHC class II*.

Next, we applied a novel systems biology approach to assess our 5x‐CC lines as a model for LOAD.^51^ A correlation analysis was performed between DEGs in 5x‐CC lines and modules associated with human AD. These 30 AD‐associated (AMP‐AD) modules were identified using human *post mortem* brain RNA‐seq data from three independent human brain transcriptome studies (ROSMAP, MSSM, and Mayo), including seven distinct brain regions, which fell into five distinct “consensus clusters.”^52^ We found that several 5x‐CC strains, and the 5x‐B6J strain, at 12 months showed a significant positive correlation (Pearson correlation ≥ 0.20; *p* < 0.05) with AD‐associated changes in several human co‐expression modules (Figure 6B). 5x‐CC013, 5x‐CC006, and 5x‐B6J displayed overlap with Consensus Cluster A, which included transcripts enriched in extracellular matrix (ECM) organization‐related pathways in the temporal cortex (TCX), inferior frontal gyrus (IFG), and parahippocampal gyrus (PHG) brain regions (modules TCXblue, IFGyellow, and PHGyellow). All 5x‐CC lines, except for 5x‐CC017, correlated with Consensus Cluster B – enriched in immune system‐related pathways in the cerebellum (CBE), superior temporal gyrus (STG), PHG, and IFG brain regions (modules CBEturquoise, STGblue, PHGturquoise, and IFGturquoise). 5x‐CC002, 5x‐CC006, 5x‐CC013, and 5x‐CC037 showed positive correlation with Cluster C – enriched in neuronal system pathways in the STG, dorsolateral prefrontal cortex (DLPFC), TCX, FP, and/or CBE brain regions (modules STGbrown, DLPFCyellow, TCXgreen, FPyellow, and CBEyellow). However, 5x‐B6J showed no positive correlation with this cluster. These data indicate that these 5x‐CC lines may be better suited to explore the specific cellular/molecular pathways (ie, neuronal) related to AD pathology than 5x‐B6J mice. In addition, all 5x‐CC lines showed a positive correlation with Consensus Cluster D – enriched in cell cycle/myelination, neuron, glia development (NMD) pathways in the DLPFC, STG, PHG, TCX, and/or IFG brain regions (modules DLPFCbrown, STGyellow, PHGgreen, TCXyellow, and IFGblue). Interestingly, 5x‐CC017 showed a significant positive correlation (Pearson correlation = 0.97; *p* < 0.05) with cell cycle/NMD changes in module CBEbrown in Consensus Cluster D but also showed a significant negative correlation with organelle biogenesis/DNA repair changes in the FPbrown module (Pearson correlation = −0.78; *p* < 0.05) and cell cycle/NMD changes in the FPblue module (Pearson correlation = −0.52; *p* < 0.05) (Figure 6B). These data suggest that CC017 may exhibit more human AD‐relevant processes in terms of myelination, neuron, and glial development but is less correlated with pathways related to organelle biogenesis and DNA repair. Of additional note, this strain has an overall lower level of correlation across the different modules, making it a less‐than‐ideal candidate for modeling human AD. Overall, these data provide evidence that, except for CC017, the CC line strains we analyzed are not drastically different from each other in terms of their overlap with human AD‐related genetic changes. Furthermore, gene expression in the 5x‐CC mice, except for 5x‐CC017, correlated with AD‐associated expression modules, suggesting that the 5x‐CC lines may emulate some of the gene expression and functional changes in ECM organization, immune system, and cell cycle/myelination, neuronal, glia development observed in the AD human brain.

Given the variance in overlap across the CC lines, we were interested in understanding the extent to which certain founder strains were contributing to AD‐related processes. To accomplish this, we explored the potential influence of founder strain(s) on this difference in overlap through assessment of founder strain contribution on AD risk genes.^14^ Using the Locus Probabilities online tool (UNC Systems Genetics), we identified locus strain information for each CC line at each AD risk gene locus and plotted this information as a heatmap (Figure 6C). We observed that the AD risk genes in C57BL/6J mice received 100% contribution from the C57BL/6J founder line, as expected, whereas AD risk genes in all the other CC lines exhibited diverse and distinctly dispersed contributions from the eight founder strains (Figure 6C). For example, CC006 displayed bimodal strain influence, receiving the highest contribution of AD risk genes from the NOD/ShiLtJ (dark blue) and 129S1/SvImJ (pink) strains. CC017 also appeared to cluster the furthest from all other mouse lines and exhibited the most equal distribution of locus information from the eight founder strains (Figure 6C). We also captured variance in founder strain contribution for each individual AD risk gene and performed hierarchical clustering based on founder strain contribution and variance. These data highlight specific AD risk genes that exhibit little variance with most contributions from a specific founder strain (ie, *Ace, Wnt3, Mapt, Grn*) as well as AD risk genes that exhibit a high level of variance in founder strain contribution (ie, *Cass4, Rbck1, Mme, Celf1, Spi1, Slc24a4*) (Figure 6C). Using these data, we can explore and dissect the role of different genetic background strains in conferring AD resilience and/or susceptibility through AD risk genes. Given that 5x‐CC006 and 5x‐CC013 displayed the highest resistance to amyloid‐related pathology and a high contribution of the NOD/ShiLtJ founder strain to AD risk genes, these data indicate that the NOD/ShiLtJ founder strain may contain genetic variations that confer resilience to AD. Examination of specific AD risk loci revealed that 5x‐CC006 and 5x‐CC013 harbored the same founder strain contributions for the following genes: *Rbck1* (PWK/PhJ), *Jazf1* (NZO/HILtj), *Tpcn1* (NZO/HILtj), *Idua* (NZO/HILtj), *Inpp5d* (129S1/SvlmJ), *Wdr81* (NOD/ShiLtJ), *Abi3* (NOD/ShiLtJ), *Ace* (NOD/ShiLtJ), *Wnt3* (NOD/ShiLtJ), *Mapt* (NOD/ShiLtJ), *Grn* (NOD/ShiLtJ), *Ctsh* (C57BL/6J), *Clu* (129S1/SvlmJ), *Ctsb* (129S1/SvlmJ), *Zcwpw1* (129S1/SvlmJ), and *Pilra* (129S1/SvlmJ). Several of these genes are involved in the regulation of cell death (*Ctsh, Grn, Ctsb, Ace, Rbck1, Mapt, Clu, Inpp5d*), metabolic processes (*Abi2, Inpp5d, Clu, Mapt, Idua, Grn, Ctsh, Ctsb, Ace, Jazf1, Tpcn1, Rbck1, Wnt3, Zcwpw1, Wdr81*), protein binding (*Abi3, Inpp5d, Clu, Mapt, Pilra, Grn, Ctsh, Ctsb, Ace, Wnt3, Zcwpw1, Wdr81, Rbck1, Tpcn1, Idua*), immune effector function (*Grn, Ctsh, Inpp5d, Clu, Ace*), and lysosomes (*Ctsh, Grn, Ctsb, Wdr81, Tpcn1, Ace, Idua*).

### 3.8 Module preservation of the CC line gene co‐expression data in MAYO dataset validates the conserved pathology revealed across diverse backgrounds

**FIGURE 7:**
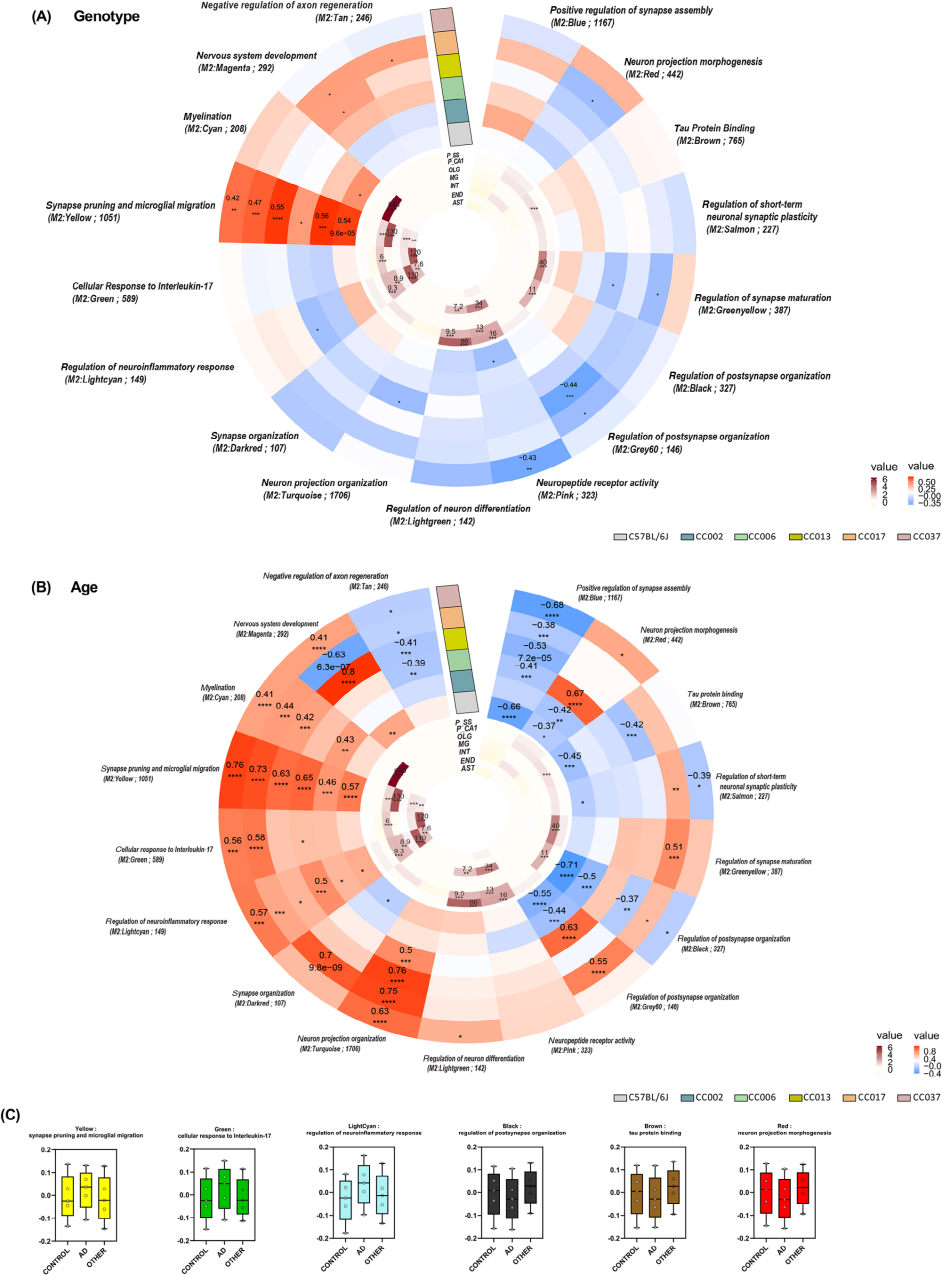
(A and B) Circular heatmaps depict consensus‐weighted gene co‐expression network built using cWGCNA, which was used to explore genotype‐ (5xFAD vs wildtype) and age‐ (4‐ vs 12‐month‐old) related changes conserved across the B6J CC F1 lines in the hippocampus, consisting of 17 gene co‐expression modules. GO‐Elite pathway analysis was performed to identify biological processes represented by each module (outer circle; red represents positive correlation, blue negative correlation; significant correlation of 0.1 and −0.1; FDR < 0.05). Cell type enrichment analysis was assessed on each module via overlap with cell type‐specific gene lists of pyramidal neurons in the somatosensory cortex (P_SS), pyramidal neurons in the CA1 (P_CA1), oligodendrocytes (OLG), microglia (MG), interneurons (INT), endothelial cells (END), and astrocytes (AST) (inner circle; dark maroon symbolizes high enrichment, pale yellow indicates no enrichment; enrichment threshold > 0.6). (C) Module preservation analysis was conducted to evaluate the preservation of our gene co‐expression network (constructed with CC line gene expression data; M2 modules) with human AD using brain MAYO RNA‐seq data. Modules that had a Z_summary_ score ≥2.0 were considered preserved (*q* < 0.05) (Figure S6). Module eigengene plots are provided for six strongly trait‐correlated modules that were also concordant with mouse changes. This analysis was conducted on all animals, including WT and 5xFAD transgene carrying mice across the different genetic backgrounds (*n* = 442 total animals; *n* = 16 for 4‐month‐old WT‐C57BL/6J, *n* = 11 for 12‐month‐old WT‐C57BL/6J; *n* = 11 for 4‐month‐old WT‐CC002, *n* = 8 for 12‐month‐old WT‐CC002; *n* = 13 for 4‐month‐old WT‐CC006, *n* = 9 for 12‐month‐old WT‐CC006; *n* = 15 for 4‐month‐old WT‐CC013, *n* = 11 for 12‐month‐old WT‐CC013; *n* = 11 for 4‐month‐old WT‐CC017, *n* = 13 for 12‐month‐old WT‐CC017; *n* = 11 for 4‐month‐old WT‐CC037, *n* = 5 for 12‐month‐old WT‐CC037; *n* = 11 for 4‐month‐old 5x‐C57BL/6J, *n* = 9 for 12‐month‐old 5x‐C57BL/6J; *n* = 14 for 4‐month‐old 5x‐CC002, *n* = 9 for 12‐month‐old 5x‐CC002; *n* = 18–19 for 4‐month‐old 5x‐CC006, *n* = 10 for 12‐month‐old 5x‐CC006; *n* = 11 for 4‐month‐old 5x‐CC013, *n* = 14 for 12‐month‐old 5x‐CC013; *n* = 13 for 4‐month‐old 5x‐CC017, *n* = 15 for 12‐month‐old 5x‐CC017; *n* = 14 for 4‐month‐old 5x‐CC037, *n* = 11 for 12‐month‐old 5x‐CC037). Statistical significance is denoted by *p < 0.05, **p < 0.01, ***p < 0.001, ****p < 0.0001.

To unravel the pathways that make C57BL/6J more susceptible to AD pathology, we performed consensus WGCNA across B6J.Cg‐5xFAD and all (B6J.Cg‐5xFAD CC)F1 strains, including all genotypes and time points. We found 17 modules containing genes related to one another by their co‐expression across genotype (Figure 7A) and age (Figure 7B) and further discerned whether these modules were enriched for specific cell types and GO pathways (GO‐Elite). This revealed several modules of interest, including the M2: Yellow module, which was the only module significantly upregulated in all 5x‐CC lines as a result of genotype. This module is associated with GO term *synapse pruning and microglial migration* and enriched in microglia. Of note, we observe that in comparing WT versus 5x, C57BL/6J, the strain with the highest level of amyloid pathology, shows the most positively significant correlation with *synapse pruning and microglial migration*, a positive correlation with M2: Cyan module *myelination* – enriched in oligodendrocytes – and a negatively significant correlation with M2: Pink module *neuropeptide receptor activity*. We also observed that 5x‐CC006, the strain with the lowest level of amyloid pathology, was the least positively correlated with *synapse pruning and microglial migration* of all the lines and exhibited a negative correlation with the M2 modules: Lightcyan *regulation of neuroinflammatory response* – enriched in endothelial cells – Turquoise *neuron projection organization*, and Greenyellow *regulation of synapse maturation* – enriched in pyramidal CA1 neurons (Figure 7A). These data indicate that the pathways that make C57BL/6J more susceptible to AD pathology could involve an upregulation in synaptic pruning, the microglial response, and myelination and a downregulation in neuropeptide receptor activity, while the pathways that make 5x‐CC006 more resistant to AD pathology could involve a less pronounced upregulation in synaptic pruning and microglial response as well as a downregulation in the neuroinflammatory response, neuron projection organization, and synapse maturation. In analyzing genetic trends as a function of age, we observed that more strains were significantly correlated to a larger portion of modules, indicating that age plays a larger role than genotype on transcriptional changes in the B6J CC F1 lines (Figure 7B). These data indicate that the majority of changes induced by aging are largely conserved across diverse backgrounds, including a negative correlation with the M2: Tan module *negative regulation of axon regeneration* and M2: Blue module *positive regulation of synapse assembly* and a positive correlation in the M2: Cyan module *myelination*, M2: Yellow module *synapse pruning and microglial migration*, M2: Green module *cellular response to Interleukin‐17*: enriched in endothelial cells, M2: Lightcyan module *regulation of neuroinflammatory response*, and M2: Turquoise module *neuron projection organization*. These data can also be used to dissect genetic variations across the CC lines within specific modules. B6 CC013 F1 is the only strain that has a positive significant correlation to the M2: Magenta module *nervous system development*, indicating there might be a unique upregulation in the genes that are associated with nervous system development in this strain, which exhibited the lowest NfL level compared to other B6J CC F1 strains. 5x‐CC006, one of the most resistant strains to AD pathology, exhibits a highly positive significant correlation in the M2: Red module *neuron projection morphogenesis*, compared to B6J, the most susceptible line to AD pathology, which exhibited a negative correlation in this module.

To examine genetic trends of this gene co‐expression network (ie, the M2 modules) with human AD, we performed module preservation using the brain MAYO RNA‐seq dataset (Figure 7C). We determined that the majority of modules are preserved with varying degrees of preservation (*q* < 0.05); modules M2: Cyan, M2: Yellow, and M2: GreenYellow are highly preserved (Z_summary_score > 10), modules M2: Turquoise, M2: Red, M2: Pink, M2: Lightgreen, and M2: Brown are preserved (Z_summary_score = 5‐10), and modules M2: Lightcyan, M2: Grey60, M2: Green, M2: Darkred, M2: Blue, and M2: Black (Z_summary_score > 2) are moderately preserved (Figure S6). We also observed that, although modules are preserved, some are discordant with mouse changes, including M2: Cyan, M2: Turquoise, and M2: Blue. The modules M2: Yellow *synapse pruning and microglial migration*, enriched for microglia, M2: Green *cellular response to Interleukin‐17*, enriched for astrocytes, and M2: Lightcyan *regulation of neuroinflammatory response*, enriched for endothelial cells, exhibit an upregulation in gene expression in both mouse and human AD samples (Figure 7C), highlighting preservation of the pathways, cells, and genes related to inflammation across species. In modules M2: Black *regulation of postsynapse organization*, enriched in pyramidal CA1 neurons, M2: Brown *tau protein binding*, enriched in pyramidal CA1 neurons, and M2: Red *neuron projection morphogenesis*, we observed a downregulation of gene expression in AD human samples compared to controls; however, this downregulation is only present in certain mouse lines. Interestingly, B6J was the only strain that exhibited a downregulation in gene expression in all three of these neuron/synaptic‐related modules, and 5x‐CC006, the most resistant strain to AD pathology, actually displayed a positive correlation or upregulation in the M2: Red module. These data may provide some insight into why certain strains may be more susceptible or resistant to AD pathology.

## 4 DISCUSSION

Accumulating evidence indicates that LOAD is a polygenic disorder influenced by multiple genetic variants.^10^, ^16^, ^18^ Efforts, including those by MODEL‐AD, are under way to develop mouse models that better recapitulate LOAD and investigate the impact of these specific AD variants on disease. An important component to consider in expanding these AD models involves the incorporation of genetic diversity.^19^ Recent developments in the generation of murine genetic reference panels and computational analysis have offered new opportunities to explore the impact of genetic diversity in mouse models. The CC genetic reference panel provides highly complex genetic diversity with >40 million variants, similar to the genetic diversity found in humans. In addition, this panel is well characterized with stable genomes that make it an indispensable tool in studying the effects of genetic diversity on disease models.^53^ Here, we combined select CC strain genomes into a well‐studied mouse model of amyloidosis, 5xFAD on a congenic C57BL/6J background, resulting in the generation of (B6J.Cg‐5xFAD x CC)F1 “5x‐CC” strains. We hypothesized that increased genetic background diversity would substantially impact the development of complex pathological traits, thereby improving the translational relevance of animal models to human AD and offering valuable insights into the pathways that regulate disease pathogenesis. The diverse CC genetic panel allowed us to examine the connections between phenotypic traits and genetic pathways that are common to both normal aging and disease. By comparing the genetic mapping results across different lines, we could identify specific genotypes that contributed to modifying a phenotype, irrespective of disease status.

Our findings reveal that introducing genetic diversity into a mouse model of AD, via the generation of 5x‐CCs, does not aggravate but instead dampens AD pathology. Here, we show that C57BL/6J congenic 5xFAD mice exhibit the highest amount of AD‐related phenotypes, including ThioS^+^ amyloid deposition, insoluble Aβ accumulation, microglial and astrocyte reactivity, and NfL. At the genetic level, congenic B6J mice exhibit the highest number of DEGs in response to aging and the introduction of the 5xFAD transgene (ie, AD) compared to other WT‐CC lines. In line with our data, previous studies showed that other inbred mouse strains (ie, DBA/2J, CE/J, 129S1/SvImJ) exhibited reduced Aβ and/or amyloid pathology compared to B6J mice.^21^, ^22^, ^24^, ^54^ Neuner et al. reported moderate Aβ levels in AD‐B6J mice relative to their AD‐BXD panel but observed an attenuation in cognitive outcomes, indicating that the B6J background strain might be resistant to cognitive changes induced by the 5xFAD transgene.^2^ Although it is possible that a decrease in Aβ/amyloid does not always result in resilience to AD, our NfL data provide strong evidence that the introduction of genetic diversity via 5x‐CC lines attenuates damage elicited in response to plaques. In line with our findings, *Htt* CAG knock‐in mice (ie, Huntington's disease model) crossed onto the B6J background presented the most rapid phenotypic progression of disease compared to those crossed with the other inbred strains.^55^ We hypothesize that, given the inbred nature of the B6J line, introduction of the 5xFAD transgene onto this line could result in an interaction with the mutations that result in exaggerated changes. Other inbred lines may provide a different palette of genetic combinations that can mute changes caused by 5xFAD transgene‐related insults or that the heterozygous alleles (or interaction of heterozygous alleles) in these lines could be protective in the 5xFAD model.

Our study also identified 5x‐CC006 as a 5x‐CC strain that exhibits heightened resistance to AD‐related processes, including reductions in ThioS^+^ and 6E10^+^ amyloid deposition, insoluble Aβ_40_ accumulation, microglial reactivity, and NfL. Moreover, 5x‐CC006 mice exhibit the fewest DEGs in response to the 5xFAD transgene. Interestingly, CC006 mice receive the highest contribution of AD risk genes from the NOD/ShiLtJ and 129S1/SvImJ strains. In addition to increased resistance to Aβ metabolism,^24^ the 129S1/SvImJ genetic background has been implicated in other models for conferring resistance to disease processes, including prolonged lifespan in Gaucher Disease – a lysosomal storage disorder.^56^ These data highlight that genetic background can influence molecular phenotypes and transcriptional responses to aging/disease, suggesting the presence of genetic modifiers, and illustrate the importance of incorporating genetic diversity in disease models. However, a larger study using more CC lines (>30 lines) is required to perform quantitative trait locus mapping to discover the variants/candidate modifiers that impact AD‐related pathologies.

Although single mouse strains are often used to establish genotype–phenotype relationships, the ability to draw conclusions about these physiological processes is based on the assumption that these relationships are also present in the human population.^57^ Our analysis reveals that distinct genetic compositions, as seen in the WT‐CC and 5x‐CC strains, can produce differential susceptibility to aging and associated biological processes, recapitulating what occurs in the human population. Furthermore, the transcriptional changes in the 5x‐CCs compared to WT‐CCs at advanced stages of disease exhibited a high level of overlap with those seen in human AD patients compared to age‐matched controls. This overlap includes an upregulation in ECM, immune, cell cycle/myelination, neuronal, and glial development related pathways, suggesting shared molecular mechanisms between mice and humans. Given the limited success in translating previous AD mouse model research into effective treatments, use of (B6J.Cg‐5xFAD CC)F1 strains represents a valuable resource for identifying modifier genes that may be more relevant to human patients. Similar to the much larger AD‐BXD panel,^2^ (B6J.Cg‐5xFAD CC)F1 strains may provide insights into the genetic basis of AD, its molecular and pathological phenotypes, and the response to interventions. However, our findings suggest that introducing eight different founder strains has a mostly modulatory effect, unlike when crossing the DBA/2J strain with B6J, which produces mice with greater pathogenic diversity.^2^ Despite this compensatory adjustment, we do observe significant variation in the glial response and functions across genotypes.

In this study, we discovered that WT‐ and 5x‐CC mice could be used not only as a tool to uncover the genetic makeup of disease resistance but also have potential in identifying genetic loci associated with glial phenotypes and functions. In evaluating the microglial response to plaques, we observed that the number of microglia in 5x‐CC mice mirrors plaque load (ie, if amyloid plaque deposition is low, then the number of microglia is also low). However, 5x‐CC037 mice displayed a significant elevation in microglia in the subiculum at 4 months despite a significant reduction in Aβ‐related deposition. A larger study using this strategy could identify genetic loci and/or molecular pathways associated with an altered microglial response to plaque pathology. A previous study comparing the B6J mouse strain with three wild‐derived mouse strains (CAST/EiJ, WSB/EiJ, and PWK/PhJ) found significant differences in the transcriptomic profile and functional diversity of microglia subtypes through WGCNA. These differences imply that natural genetic variation may affect the initial microglial response to plaques, response efficiency, and return to surveillance/homeostatic state, which was supported by our study's findings. Additionally, the same wild‐derived strains exhibit significantly fewer cortical and hippocampal ThioS+ plaques compared to B6J when crossed with Aβ generating lines.^58^ 5x‐CC017 mice showed a significant reduction in astrocytes, specifically in cortical GFAP^+^ astrocytes at both 4 and 12 months. Studies indicate that A1 reactive astrocytes, activated by microglia, may be responsible for inducing neuronal death.^59^ However, no decrease in cortical NfL was observed in these 5x‐CC017 mice. Correlation analysis reveals that the 5x‐CC017 strain also exhibits the least amount of overlap with the human AD co‐expression module associated with the immune system. Further investigation is needed to better understand the genetic makeup of this strain and the potential genetic modifiers that could lead to this altered response of reactive astrocytes. Given these disparate glial responses across the CC lines used, we believe the strategy of using (B6J.Cg‐5xFAD CC)F1 strains is suitable for uncovering the genetic associations to differences in biological processes associated with Aβ‐related pathologies, including glial responses. Our findings also suggest that the B6J inbred strain may be the most susceptible to plaque formation and can serve as an appropriate genetic background model for simulating AD, as it enables the development of plaques, along with corresponding glial and gene expression changes in response to these plaques, and generates the highest level of NfL, indicative of damage.

Despite our ability to dissect the contributions of Aβ‐related pathways on AD pathogenesis in 5x‐CC mice, the 5x‐CC lines also harbor the same caveats as traditional transgenic mice. These include overexpression of transgenic APP and PS1 proteins, loss or disruption of endogenous gene expression, mRNA splicing, and/or chromosomal loci by insertion of transgenic construct, variable expression of transgene, timing of transgene expression that may not mimic disease, and lack of tau pathology.^20^, ^60^, ^61^ Although the CC strains we used were selected based on their diversity in expression of genes known to be associated with AD risk, it is possible that in the generation of the F1 crosses using B6J.5xFAD hemizygous mice crossed to the CC lines, expression of such risk genes might no longer be the same as in the original CC lines. Future mouse models could address these concerns by crossing recombinant inbred CC strains with AD mouse models that contain humanized *APP* sequences with LOAD risk variants for improved clinical recapitulation of human AD. However, current humanized AD models lack apparent amyloid or tau pathology, making it difficult to assess the roles of these variants and/or genetic diversity on classic hallmarks of AD. Future studies that could be of benefit are currently under way in MODEL‐AD to investigate the role of environmental factors (ie, diet) on the development of AD in humanized models of LOAD, which will have important implications for the development of future therapeutic strategies.

## CONFLICT OF INTEREST STATEMENT

The authors declare no conflicts of interest. Author disclosures are available in the supporting information.

## DISCLOSURES

KNG is a member of the advisory board of Ashvattha Therapeutics. The other authors have nothing to disclose.

## CONSENT STATEMENT

Human data were obtained from the AD Knowledge Portal. No human subjects were used for the present study. Therefore, consent was not necessary.

## Supporting information

Supporting Information

Supporting Information
