# Supplementary material for: Genetic diversity promotes resilience in a mouse model of Alzheimer's disease
Source: Alzheimers Dement. 2024 Mar 1;20(4):2794–816. doi: 10.1002/alz.13753 (PMC11032575; doi:10.1002/alz.13753)
Supplement: Supplementary file 2 — Supporting Information [file ALZ-20-2794-s001.pdf]

Sex

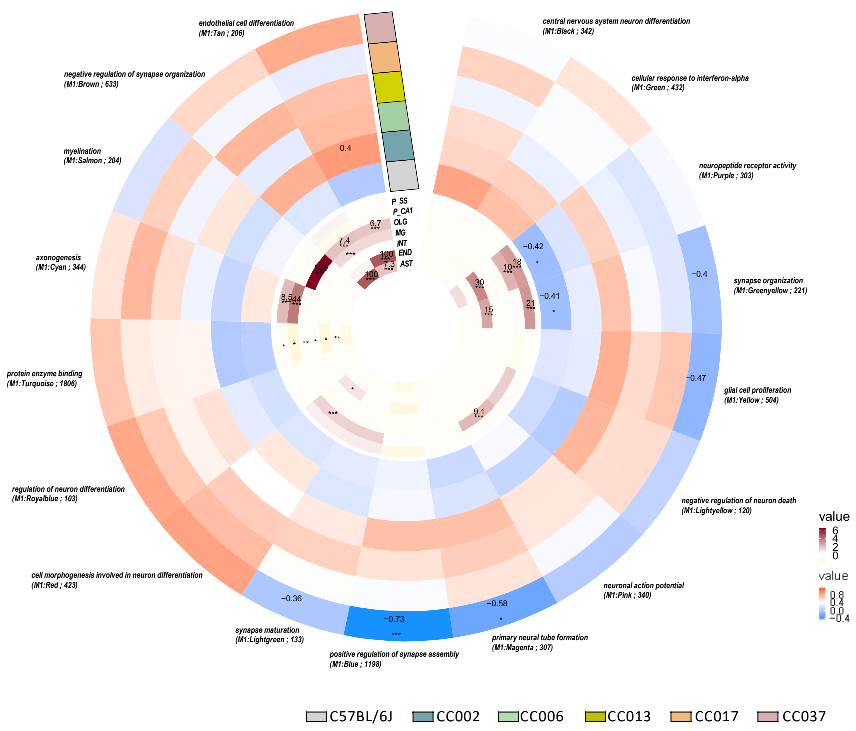

Supplemental Figure 1

Supplemental Fig. 1: A circular heatmap depicts the consensus weighted gene co-expression network built using cWGCNA, which was used to explore sex-related changes (male vs female) conserved across the WT-CC lines in the hippocampus, consisting of 17 gene co-expression modules. GO-Elite pathway analysis was performed to identify biological processes represented by each module (outer circle; red represents positive correlation and blue represents negative correlation; significant correlation of 0.1 and -0.1; FDR < 0.05). Cell type enrichment analysis was assessed on each module via overlap with cell type-specific gene lists of pyramidal neurons in the somatosensory cortex (P_SS), pyramidal neurons in the CA1 (P_CA1), oligodendrocytes (OLG), microglia (MG), interneurons (INT), endothelial cells (END), and astrocytes (AST) (inner circle; dark maroon symbolizes high enrichment and pale-yellow shows no enrichment; enrichment threshold > 0.6). Statistical significance is denoted by *$p<0.05$, **$p<0.01$, ***$p<0.001$, ****$p<0.0001$.

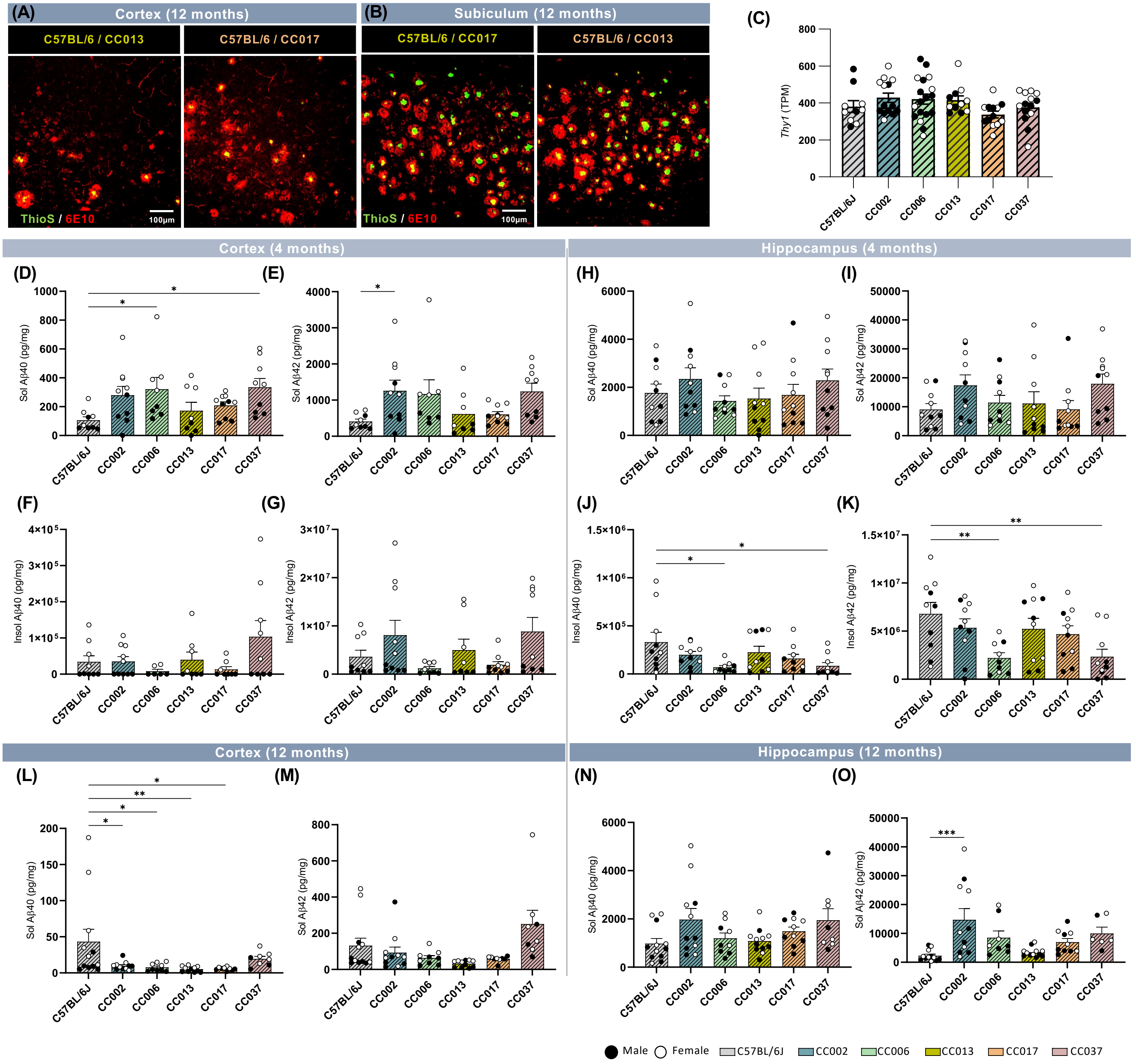

Supplemental Figure 2

Supplemental Fig. 2: (A-B) Representative confocal images of the (A) somatosensory cortex and (B) subiculum in 12-month-old 5xFAD transgene carrying F1 progeny of CC mice (5x-(B6J CC013)F1 and 5x-(B6J CC017)F1) immunolabeled for immunolabeled for ThioS (green) and 6E10 (red). Scale bar = 100µm. **(C)** *Thy1* expression quantified by number of transcripts per million reads (TPM) in 4-month-old 5x-CC mice in the hippocampus (n=77 mice across five strains). **(D-O)** Quantification of soluble (D-E, H-I, L-O) and insoluble (F-G, J-K) Aβ40 and Aβ42 protein in the cortex (D-E, F-G, L-M) and hippocampus (H-I, J-K, N-O) in 4- (D-K) and 12-month-old (L-O) 5x-B6J and (5x-B6J CC)F1 lines (5x-CC002, 5x-CC006, 5x-CC013, 5x-CC017, 5x-CC037). Data are represented as mean ± SEM. Statistical analysis was performed using a one-way ANOVA with Dunnett test. *$p<0.05$, **$p<0.01$, ***$p<0.001$, ****$p<0.0001$.

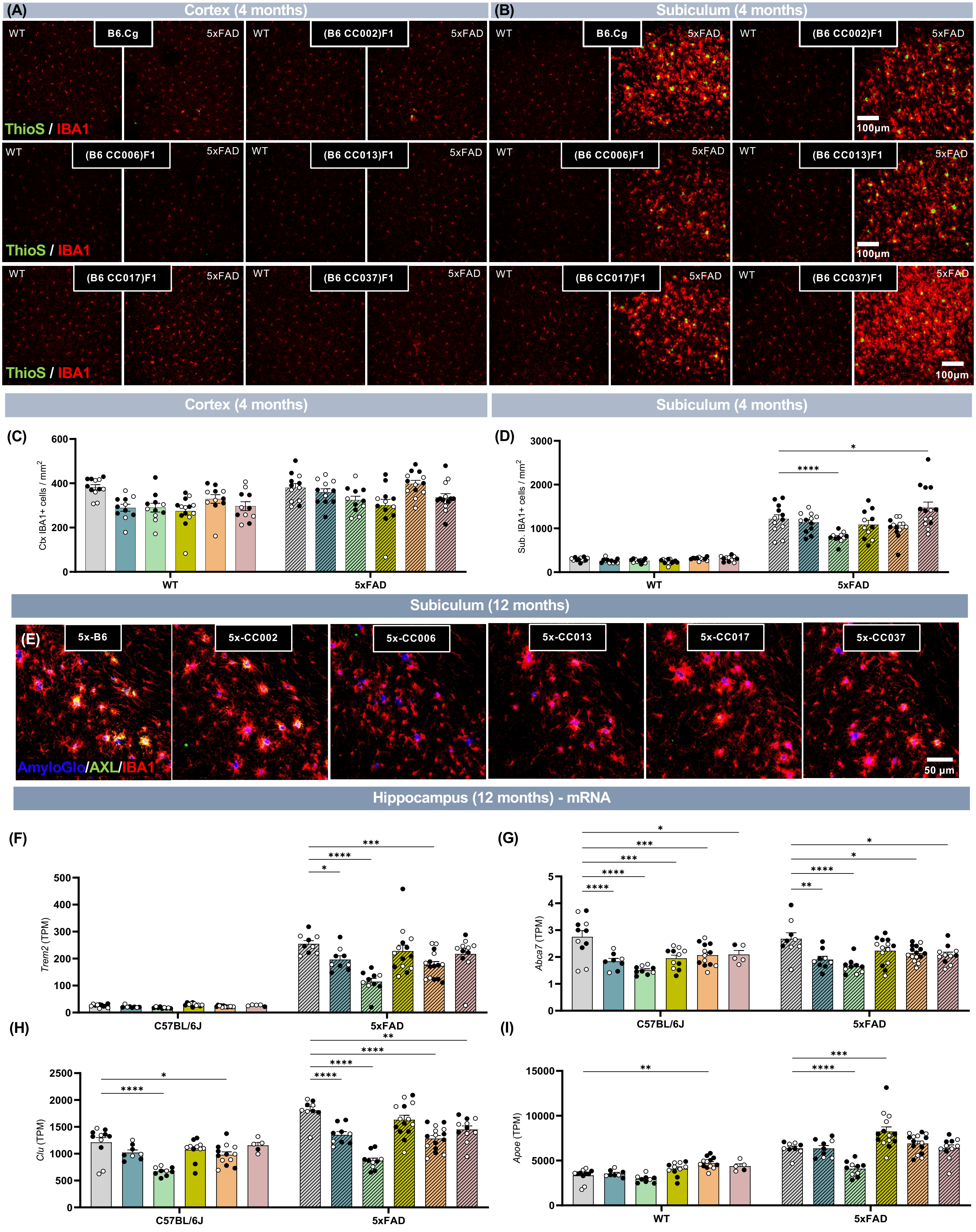

Supplemental Figure 3

Supplemental Fig. 3: (**A-B**) Representative confocal images of the somatosensory cortex and subiculum in 4-month-old wildtype (WT) and 5xFAD transgene carrying F1 progeny of CC mice (WT-B6J, 5x-B6J, WT- (B6J CC002)F1, 5x-(B6J CC002)F1,WT-(B6J CC006)F1, 5x-(B6J CC006)F1, WT-(B6J CC013)F1, 5x-(B6J CC013)F1, WT-(B6J CC017)F1, and 5x-(B6J CC017)F1, WT-(B6J CC037)F1, and 5x-(B6J CC037)F1) immunolabeled for Thioflavin-S (Thio-S, green) and microglia (IBA1, red). Scale bar = 100µm. (**C-D**) Quantification of microglia per mm^2 in the cortex (C) and subiculum (D) at 4 months. **(E)** Representative confocal images of the subiculum in 12-month-old 5x-B6J and 5x-CC lines immunolabeled for plaques (AmyloGlo, blue), microglia (IBA1, red), and a marker for microglial activation (AXL, green). (**F-I**) Expression of microglial and AD-related genes quantified by number of transcripts per million reads (TPM) in 12-month-old WT-CC and 5x-CC mice in the hippocampus (n=125 mice across ten lines)**.** Data are represented as mean ± SEM. Statistical analysis was performed using a two-way ANOVA with Dunnett test. *p<0.05, **p<0.01, ***p<0.001, ****p<0.0001.

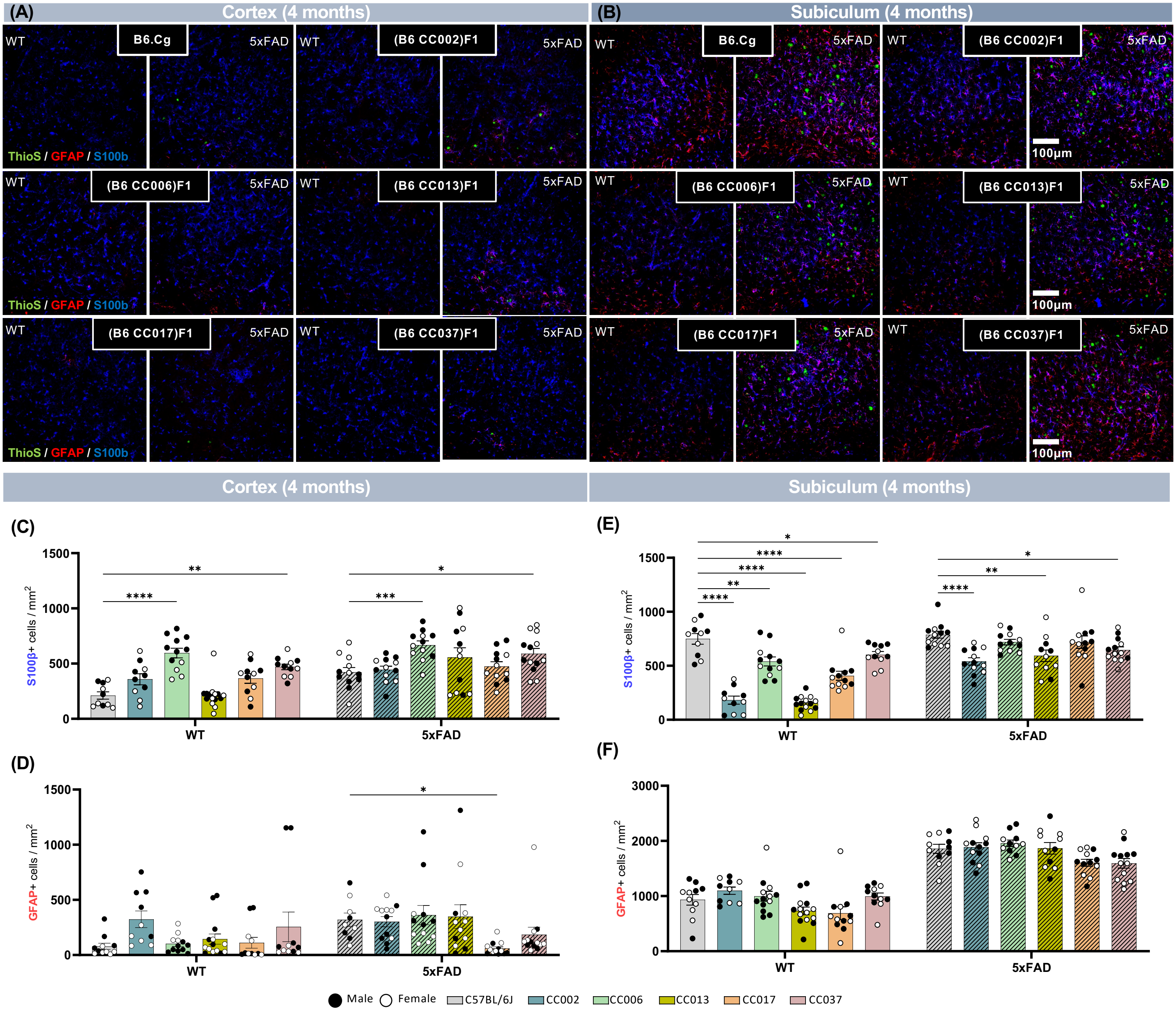

Supplemental Figure 4

Supplemental Fig. 4: (**A-B**) Representative confocal images of the somatosensory cortex and subiculum in 4-month-old wildtype (WT) and 5xFAD transgene carrying F1 progeny of CC mice (WT-B6J, 5x-B6J, WT- (B6J CC002)F1, 5x-(B6J CC002)F1,WT-(B6J CC006)F1, 5x-(B6J CC006)F1, WT-(B6J CC013)F1, 5x-(B6J CC013)F1, WT-(B6J CC017)F1, and 5x-(B6J CC017)F1, WT-(B6J CC037)F1, and 5x-(B6J CC037)F1) immunolabeled for Thioflavin-S (Thio-S, green), reactive astrocytes (GFAP, red) and all astrocytes (S100β, blue). Scale bar = 100µm. (**C-F**) Quantification of S100β^+ (C, E) and GFAP$^+$ (D, F) astrocytes per mm^2 in the cortex (C-D) and subiculum (E-F) at 4 months of age. Data are represented as mean ± SEM. Statistical analysis was performed using a two-way ANOVA with Dunnett test. *$p<0.05$, **$p<0.01$, ***$p<0.001$, ****$p<0.0001$.

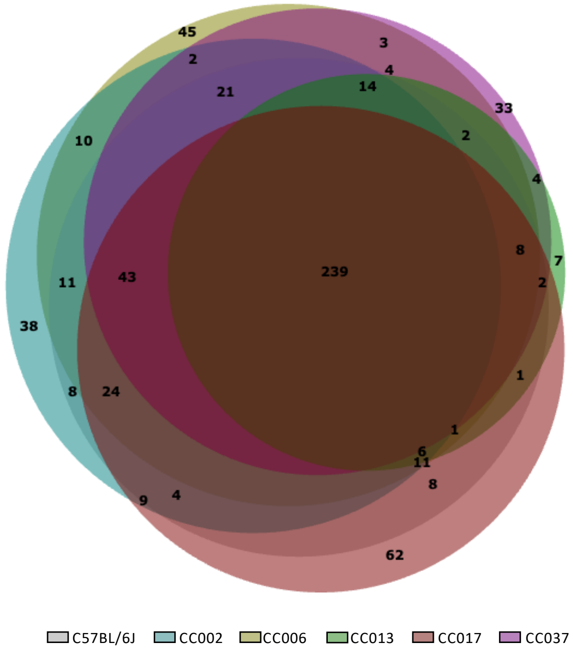

Supplemental Figure 5

Supplemental Fig. 5: Venn diagram showing the overlap in conserved DEGs (5xFAD vs WT and 5x-CCs vs WT-CCs) across the different strains.

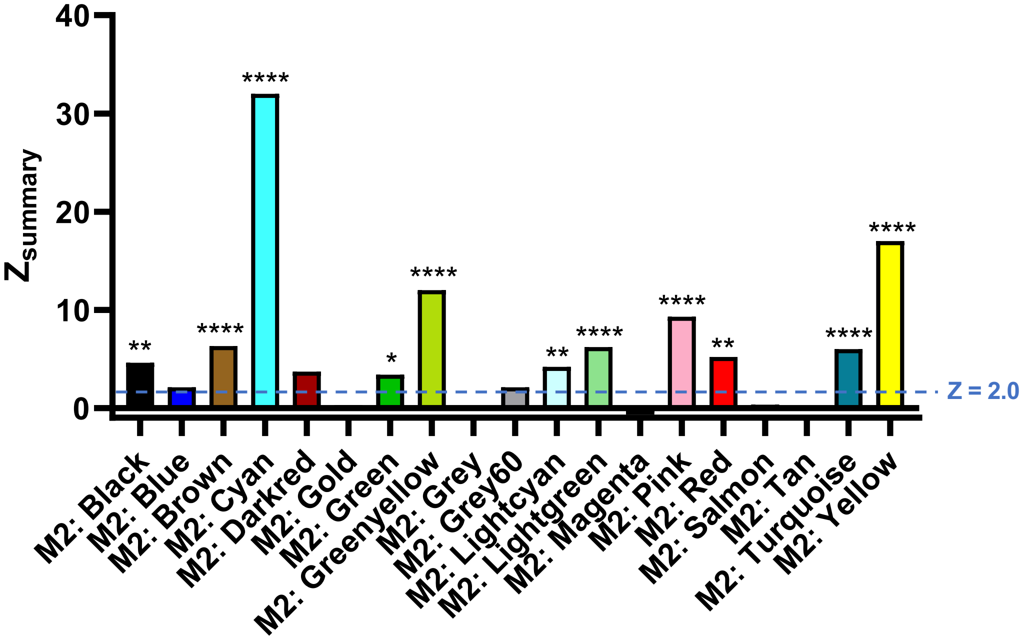

Supplemental Figure 6

Supplemental Fig. 6: Module preservation analysis was conducted to evaluate the preservation of our gene co-expression network (constructed with CC line gene expression data; M2 modules) with human AD using brain MAYO RNAseq data. Modules that had a $Z_{summary}$ score greater than or equal to 2.0 were considered preserved (blue dotted line; q<0.05). This analysis was conducted on all animals, including wildtype and 5xFAD transgene carrying mice across the different genetic backgrounds (n=442). Preservation statistical analysis was performed using a Bonferroni test. *q<0.05, **q<0.01, ***q<0.001, ****q<0.0001.
